# Reduced Plasma Levels of 25-Hydroxycholesterol and Increased Cerebrospinal Fluid Levels of Bile Acid Precursors in Multiple Sclerosis Patients

**DOI:** 10.1007/s12035-016-0281-9

**Published:** 2016-11-23

**Authors:** Peter J. Crick, William J. Griffiths, Juan Zhang, Martin Beibel, Jonas Abdel-Khalik, Jens Kuhle, Andreas W. Sailer, Yuqin Wang

**Affiliations:** 1 Swansea University Medical School, Singleton Park, Swansea, SA2 8PP UK; 2 Analytical Science and Imaging, Novartis Institutes for BioMedical Research, CH-4002 Basel, Switzerland; 3 Developmental & Molecular Pathways, Novartis Institutes for BioMedical Research, CH-4002 Basel, Switzerland; 4 Neurology, Departments of Medicine, Biomedicine and Clinical Research, University Hospital Basel, CH-4031 Basel, Switzerland

**Keywords:** Sterol, Oxysterol, Bile acid, CNS, Inflammation, HPLC, *MS*

## Abstract

Multiple sclerosis (MS) is an autoimmune, inflammatory disease of the central nervous system (CNS). We have measured the levels of over 20 non-esterified sterols in plasma and cerebrospinal fluid (CSF) from patients suffering from MS, inflammatory CNS disease, neurodegenerative disease and control patients. Analysis was performed following enzyme-assisted derivatisation by liquid chromatography–mass spectrometry (LC–*MS*) exploiting multistage fragmentation (*MS* ^*n*^). We found increased concentrations of bile acid precursors in CSF from each of the disease states and that patients with inflammatory CNS disease classified as suspected autoimmune disease or of unknown aetiology also showed elevated concentrations of 25-hydroxycholestertol (25-HC, *P* < 0.05) in CSF. Cholesterol concentrations in CSF were not changed except for patients diagnosed with amyotrophic lateral sclerosis (*P* < 0.01) or pathogen-based infections of the CNS (*P* < 0.05) where they were elevated. In plasma, we found that 25-HC (*P* < 0.01), (25R)26-hydroxycholesterol ((25R)26-HC, *P* < 0.05) and 7α-hydroxy-3-oxocholest-4-enoic acid (7αH,3O-CA, *P* < 0.05) were reduced in relapsing-remitting MS (RRMS) patients compared to controls. The pattern of reduced plasma levels of 25-HC, (25R)26-HC and 7αH,3O-CA was unique to RRMS. In summary, in plasma, we find that the concentration of 25-HC in RRMS patients is significantly lower than in controls. This is consistent with the hypothesis that a lower propensity of macrophages to synthesise 25-HC will result in reduced negative feedback by 25-HC on IL-1 family cytokine production and exacerbated MS. In CSF, we find that the dominating metabolites reflect the acidic pathway of bile acid biosynthesis and the elevated levels of these in CNS disease is likely to reflect cholesterol release as a result of demyelination or neuronal death. 25-HC is elevated in patients with inflammatory CNS disease probably as a consequence of up-regulation of the type 1 interferon-stimulated gene *cholesterol 25-hydroxylase* in macrophages.

**Electronic supplementary material:**

The online version of this article (doi:10.1007/s12035-016-0281-9) contains supplementary material, which is available to authorized users.

## Introduction

Multiple sclerosis (MS) is an autoimmune inflammatory disease of the central nervous system (CNS). A large number of genetic variants, together with several putative environmental agents, including serum vitamins D levels, Epstein–Barr virus infection and a history of smoking, determine MS susceptibility [1, 2]. The disease in most cases follows a relapsing–remitting (RR) course where acute autoimmune attacks against the CNS are followed by recovery. Many patients with RRMS go on to develop secondary progressive MS, characterised by irreversible neurological disability. Immunological and pathological studies have provided evidence supporting an important role played by the immune system in driving the abnormal demyelinating process seen in MS patients [3, 4]. Neuronal degeneration is also a key in MS pathogenesis and is present already in early disease stages and is especially evident in the progressive phase of the disease, when brain atrophy and irreversible disability are more prominent [4, 5]. Type 1 interferon (IFN) is used in the treatment of MS, it has a suppressive effect on immunity [6]. IFN-stimulated genes include *cholesterol 25-hydroxylase* (*CH25H*) which is up-regulated in macrophages upon bacterial or virus infection [7–10]. Recently, several disease-modifying drugs (DMDs) in addition to the first generation of injectable DMDs (IFN and glatiramer acetate) have been developed and licensed. These include natalizumab, fingolimod, dimethyl fumarate, teriflunomide and alemtuzumab.

Perturbation of sterol and cholesterol pathways has recently been linked to various immune disorders [11, 12]. Oxysterols, oxidised metabolites of cholesterol or its precursors, are key mediators of these pathways. As well as being essential metabolites controlling cholesterol levels and leading to the production of bile acids, oxysterols have been shown to modulate the immune system. They, and their downstream metabolites, are ligands for nuclear hormone receptors such as the liver X receptors (LXRs), the farnesoid X receptor, the pregnane X receptor, the RAR-related orphan receptor γt (RORc2) [13–16], they modulate transcription in macrophages [17], and RORc2 activation plays a central role in the differentiation of T_h_17 cells [18]. Furthermore, oxysterols can activate a G protein-coupled receptor called Epstein–Barr virus-induced gene 2 (EBI2, GPR183) and oxysterol gradients guide migration of EBI2 expressing immune cells [19, 20], many of which have been implicated in shaping the adapted and innate immune response. We hypothesised that oxysterol concentrations vary under pathophysiological conditions and set out to determine using liquid chromatography–mass spectrometry (LC–*MS*) oxysterol levels in plasma and cerebrospinal fluid (CSF) from patients suffering from MS, both RRMS and clinically isolated syndrome (CIS), and from symptomatic control patients (CP). Note, we use italics to differentiate the abbreviation for mass spectrometry (*MS*), from that for multiple sclerosis (MS). In addition, samples from patients with neurodegenerative disease i.e. Alzheimer’s disease (AD) or Parkinson’s disease (PD), amyotrophic lateral sclerosis (ALS), and inflammatory CNS disease i.e. suspected autoimmune disease or of unknown aetiology (SA/UA) and pathogen-based infection (PBI), were analysed. The CSF data for the CP group has been published elsewhere [21].

## Materials and Methods

### Patients and Controls

Written informed consent was obtained from all patients in accordance with the Declaration of Helsinki, and the study was approved by the Common Institutional Review Board of the Cantons of Basel, Switzerland.

Samples were stratified into the following groups: (1) CIS, (*n* = 16); (2) RRMS, (*n* = 17); (3) CP (*n* = 18), i.e. patients with neurological symptoms, but no objective clinical or paraclinical findings to define a specific neurological disease at the time of sampling (CSF negative for oligoclonal bands, normal blood brain barrier function, and normal cell count); (4) inflammatory CNS disease subdivided into (4.1) SA/UA (*n* = 10); and (4.2) PBI (*n* = 9); and (5) neurodegenerative diseases made up of (5.1) AD and PD (*n* = 9); and (5.2) ALS (*n* = 11). The Table shown in Online Resource 1 summarises patient information.

### Lipid Extraction and LC–*MS*

Non-esterified sterols in plasma were assayed by LC–*MS* exploiting enzyme-assisted derivatisation utilising the Girard P (GP) reagent as illustrated in the Fig. in Online Resource 2. The method is fully described in [22]. For CSF analysis, the only modifications made to the published protocol for plasma were that the volume of CSF used was 250 μL, while that for plasma was 100 μL; the concentrations of internal standards were 16 ng/mL of 24R/S-[25,26,26,26,27,27,27-^2^H_7_]hydroxycholesterol, 1.6 ng/mL of 7α,25-[26,26,26,27,27,27-^2^H_6_]dihydroxycholesterol, 16 ng/mL of 22R-[25,26,26,26,27,27,27-^2^H_7_]hydroxylcholest-4-en-3-one and 16 μg/mL of [25,26,26,26,27,27,27-^2^H_7_]cholesterol. The size of the final C_18_ column used for CSF was 50 mg while that used for plasma was 200 mg. Exact details are given in Online Resource 3.

### Statistical Analysis

**Fig. 1:**
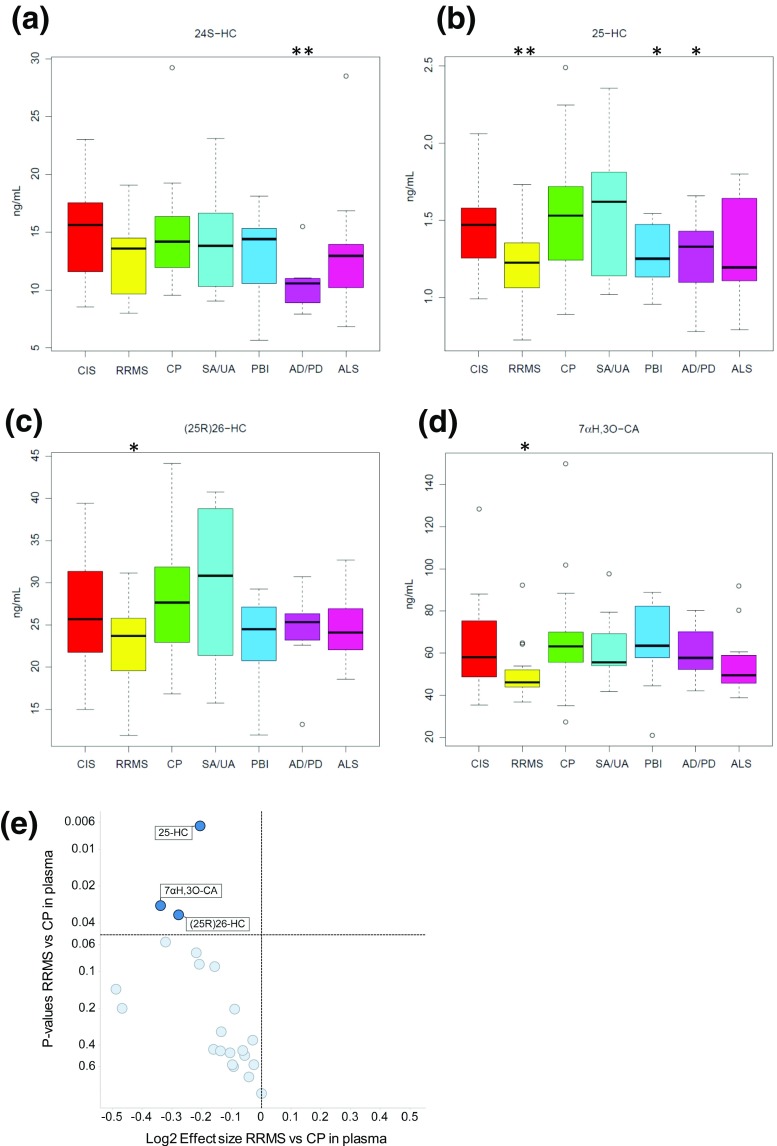
Effect of CNS disease on sterol concentrations in plasma. *Box* and *whiskers plots* showing the concentrations (ng/mL) of **a** 24S–HC, **b** 25-HC, **c** (25R)26-HC, and **d** 7αH,3O–CA in plasma from CIS (*n* = 16), RRMS (*n* = 17), CP (*n* = 18), SA/UA (*n* = 10), PBI (*n* = 9), AD/PD (*n* = 9) and ALS (*n* = 11) patients. Uni-variant *t* tests were performed against the CP group, *P < 0.05; **P < 0.01. **e** Volcano plot for plasma data showing the *P* value versus base2 logarithm of fold change for the RRMS group against CP

**Fig. 2:**
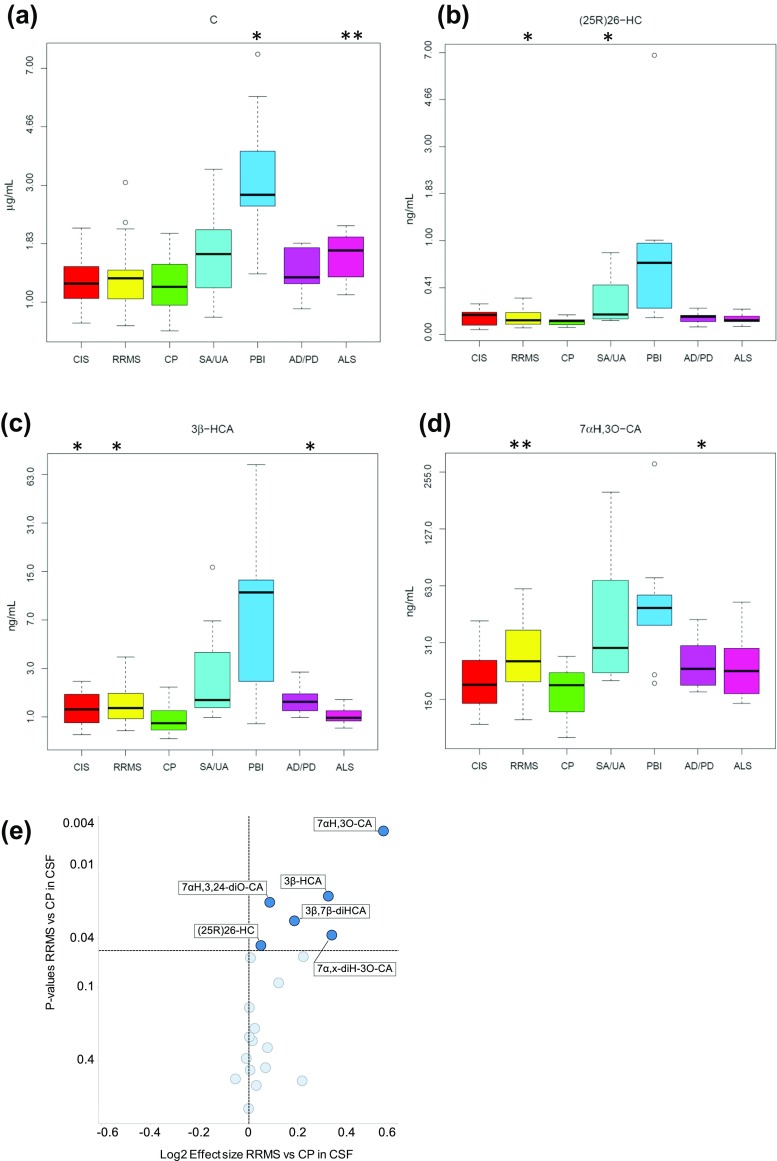
Effect of CNS disease on sterol concentrations in CSF. *Box* and *whiskers plots* showing the concentrations of **a** cholesterol, **b** (25R)26-HC, **c** 3β-HCA and **d** 7αH,3O-CA in CSF from CIS (*n* = 16), RRMS (*n* = 17), CP (*n* = 18), SA/UA (*n* = 10), PBI (*n* = 9), AD/PD (*n* = 9) and ALS (*n* = 11) patients. Cholesterol concentration is in microgram per milliliter other analyte concentrations are in nanogram per milliliter. To facilitate visualisation, the *y*-axis is on a log2 scale. Uni-variant *t* tests were performed against the CP group, *P < 0.05; **P < 0.01. **e** Volcano plot for CSF data showing the *P* value versus base2 logarithm of fold change for the RRMS group against CP

An ANOVA test was run for each sterol on linear and logarithmic scales. The log scale used a transformation log2(+1) to avoid issues with zero and small numbers. Uni-variant *t* tests were performed against the CP group, *P < 0.05; **P < 0.01. Concentrations given in the text are mean ± standard deviation (SD). The boxplots in Figs. 1 and 2 and the Figs. in Online Resources 4 and 5 were generated with default parameters in R version 3.02. The bottom and top of the central box are the first and third quartiles, and the band inside the box is the median. The whiskers extend to the most extreme data points which are no more than 1.5 times the range between the first and third quartile distant from the box. Points beyond that are plotted individually. Pair-wise correlations between CSF or plasma levels and specific analyte were performed by R version 3.02. *P* values for the significance of the correlations are listed in the Tables in Online Resources 6 and 7, respectively. The *P* values that are below 0.05/((21*20)/2) = 0.000238 for CSF and below 0.05/((22*21)/2) = 0.000216 for plasma are highlighted in the Online Resource Tables, these are significant after a Bonferronni correction at 5 %.

The volcano plots of statistical significance (*P* values) against log2 fold change between RRMS and CP shown in Figs. 1e and 2e, demonstrating the most significantly differentially regulated metabolites, were generated with TIBCO Spotfire (TIBCO Software) using log2(+1) transformation. The *t* test was made using the modification by Welch that accommodates for different variances in the two groups.

## Results

### Plasma

**Fig. 3:**
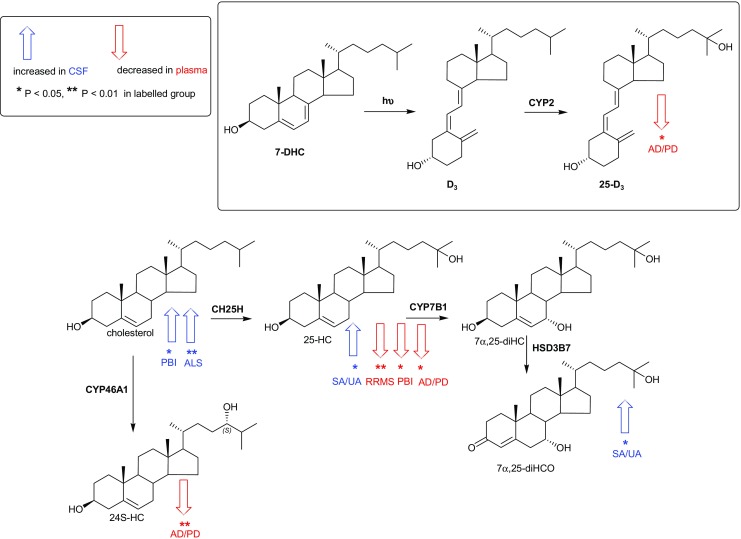
Sterol metabolism via the cholesterol 24S- and 25-hydroxylase pathways in CNS. Changes in sterol concentrations in CSF and plasma are indicated by *blue* and *red arrows,* respectively. The direction of change corresponds to the direction of the arrow. Enzyme abbreviations used are *CH25H* cholesterol 25-hydroxylase, *CYP* cytochrome P450, *HSD* hydroxysteroid dehydrogenase

Shown in Fig. 1a–d and in the Fig. in Online Resource 4 are box and whisker plots showing the concentrations of non-esterified oxysterols, 25-hydroxyvitamin D_3_ (25-D_3_), cholestenoic and cholenoic acids in plasma. Table 1 lists the sterols analysed and the Table in Online Resource 8 gives the measured concentrations for each patient group. In the RRMS samples, the concentration of 25-hydroxycholesterol (25-HC) is reduced significantly compared to CP (1.19 ± 0.26 ng/mL cf. 1.54 ± 0.42 ng/mL, mean ± SD, *P* < 0.01). We also see a reduction in the concentration of (25R)26-hydroxycholesterol ((25R)26-HC, 22.63 ± 5.03 ng/mL cf. 27.97 ± 7.89 ng/mL, *P* < 0.05) and 7α-hydroxy-3-oxocholest-4-enoic acid (7αH,3O-CA, 50.60 ± 13.33 ng/mL cf. 66.88 ± 26.93 ng/ml *P* < 0.05) in RRMS patients. It is important to note that 25-HC is generated by macrophages [7, 9, 23], while the latter two compounds are synthesised by multiple different cell types, making their exact origin in plasma difficult to assess [24]. The levels of the 25-HC metabolites 7α,25-dihydroxycholesterol (7α,25-diHC) and 7α,25-dihydroxycholest-4-en-3-one (7α,25-diHCO) in RRMS plasma do not differ from CP values (0.22 ± 0.08 ng/mL and 1.06 ± 0.33 ng/mL, respectively, Fig. 3). This data suggests that the fall in 25-HC concentration in RRMS patients is a consequence of reduced production of 25-HC arising from either reduced transcription/translation of *CH25H* or reduced activity of the enzyme and that RRMS patients have a reduced capacity to produce 25-HC.

**Table 1:** Oxysterols, cholestenoic and cholenoic acids and vitamin D_3_ metabolites analysed by LC–*MS* in the present study. Concentrations measured in plasma and CSF are given in Online Resources 8 and 9, respectively

Sterol systematic name (common name)	Lipid maps ID	Abbreviation	Code
9,10-Secocholesta-5Z,7E,10-trien-3β,25-diol (25-Hydroxyvitamin D_3_)	LMST03020246	25-D_3_	C_1
Cholest-5-en-3β-ol (cholesterol)	LMST01010001	C	C_2
Cholest-4-ene-3β,6-diol or Cholest-5-ene-3β,6-diol (6-hydroxycholesterol)	–	6-HC	C_3
Cholest-5-ene-3β,7α-diol (7α-hydroxycholesterol)	LMST01010013	7α-HC	C_4
7α-Hydroxycholest-4-en-3-one	LMST04030123	7α-HCO	C_5
Cholest-5-ene-3β,7β-diol (7β-hydroxycholesterol)	LMST01010047	7β-HC	C_6
3β-Hydroxycholest-5-en-7-one (7-oxocholesterol)	LMST01010049	7O-C	C_7
Cholest-5-ene-3β,24S-diol (24S-hydroxycholesterol)	LMST01010019	24S-HC	C_8
Cholest-5-ene-3β,25-diol (25-hydroxycholesterol)	LMST01010018	25-HC	C_9
Cholest-5-ene-3β,(25R)26-diol ((25R)26-hydroxycholesterol)	LMST01010088	(25R)26-HC	C_10
Cholest-5-ene-3β,7α,25-triol (7α,25-Dihydroxycholesterol)	LMST04030166	7α,25-diHC	C_11
7α,25-Dihydroxycholest-4-en-3-one	LMST04030107	7α,25-diHCO	C_12
Cholest-5-ene-3β,7α,(25R)26-triol (7α,(25R)26-dihydroxycholesterol)	LMST04030081	7α,(25R)26-diHC	C_13
7α,(25R)26-Dihydroxycholest-4-en-3-one	LMST04030157	7α,(25R)26-diHCO	C_14
3β-Hydroxycholest-5-enoic acid	LMST04030072	3β-HCA	C_15
3-Oxocholest-4-enoic acid	LMST04030217	3O-CA	C_16
3β,7β-Dihydroxycholest-5-enoic acid	–	3β,7β-diHCA	C_17
3β,7α-Dihydroxycholest-5-enoic acid	LMST04030148	3β,7α-diHCA	C_18
7α-Hydroxy-3-oxocholest-4-enoic acid	LMST04030149	7αH,3O-CA	C_19
7α,x-Dihydroxy-3-oxocholest-4-enoic acid	–	7α,x-diH,3O-CA	C_20
7α,y-Dihydroxy-3-oxocholest-4-enoic acid	–	7α,y-diH,3O-CA	C_21
7α-Hydroxy-3,24-*bis*oxocholest-4-enoic acid	–	7αH,3,24-diO-CA	C_22
7α-Hydroxy-26-*nor*-cholest-4-ene-3,24-dione	–	7αH,26-nor-C-3,24-diO	C_23
3β-Hydroxychol-5-enoic acid	LMST04010201	3βH-Δ^5^-BA	C_24
3β,7α-Dihydroxychol-5-enoic acid	LMST04010217	3β,7α-diH-Δ^5^-BA	C_25
7α-Hydroxy-3-oxochol-4-enoic acid	LMST04010239	7αH,3O-Δ^4^-BA	C_26

Note that we use the systematic nomenclature where addition of a hydroxy group to the terminal side chain of cholesterol leading to R stereochemistry at C-25 gives the compound (25R)26-hydroxycholesterol. In much of the literature, this compound is known by the non-systematic name 27-hydroxycholesterol

In agreement with some, but not all, earlier studies, the level of 24S-hydroxycholesterol (24S-HC) is reduced in plasma from the group of patients with either AD or PD compared to CP (10.51 ± 2.18 ng/mL cf. 14.99 ± 4.57 ng/mL, *P* < 0.01) [25, 26]. The level of 25-HC is also reduced in plasma of AD/PD patients (1.26 ± 0.28 ng/mL, *P* < 0.05), as it is in patients diagnosed with PBI (1.28 ± 0.21 ng/mL, *P* < 0.05), compared to CP (1.54 ± 0.42 ng/mL).

### CSF

**Fig. 4:**
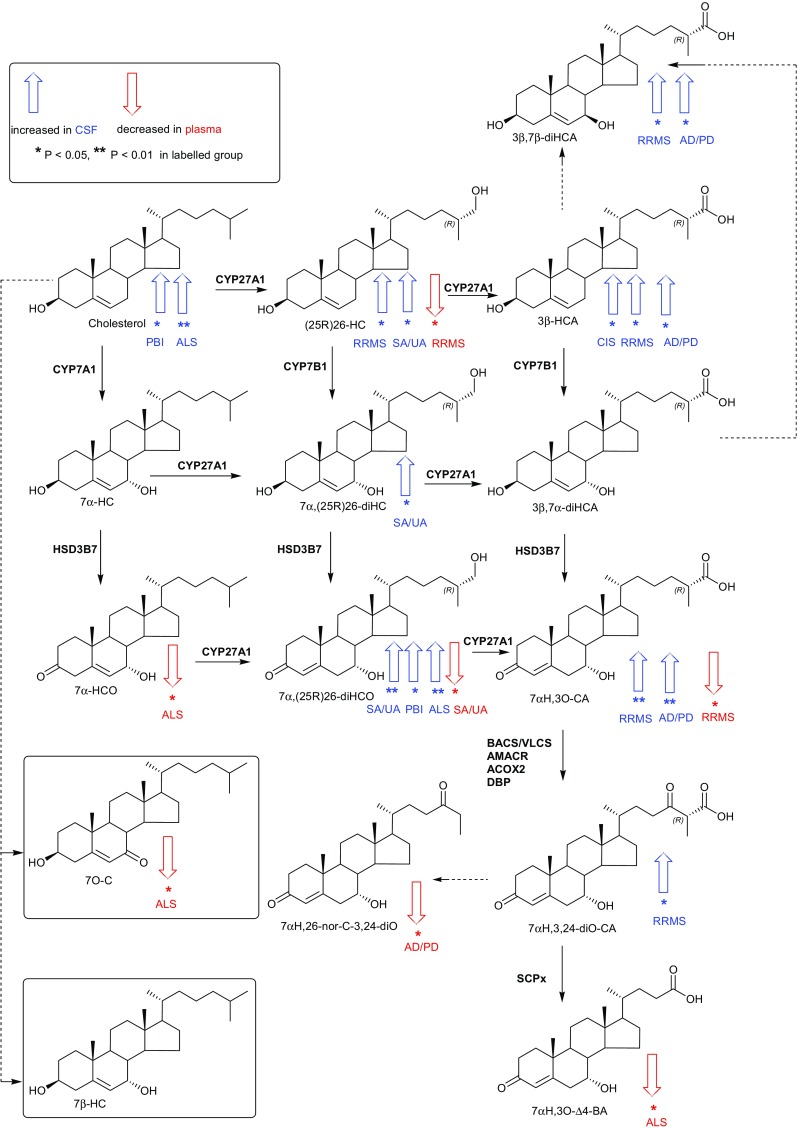
Sterol metabolism via the bile acid biosynthesis pathways in CNS. The acidic pathway starts with (25R)26-hydroxylation of cholesterol by CYP27A1, the neutral pathway with 7α-hydroxylation of cholesterol by CYP7A1. Changes in sterol concentration in CSF and plasma are indicated by *blue* and *red arrows,* respectively. The direction of change corresponds to the direction of the arrow. Enzyme abbreviations used are *ACOX2* acyl-CoA oxidase 2, branched chain, *AMACR* alpha-methylacyl-CoA racemase, *BACS* bile acyl-CoA synthetase, *DBP* D-bifunctional protein or multifunctional enzyme type 2 (HSD17B4), *SCPx*, sterol carrier protein x; *VLCS* very long chain acyl-CoA synthetase

It is generally considered that in healthy individuals CSF levels of (25R)26-HC correlate with levels of this oxysterol in the circulation [27]. However, this is not necessarily the case with (25R)26-HC in CSF from patients with neurodegenerative diseases [28]. In our study, we find patients with RRMS, in contrast to our observations in plasma, show an increase in the concentration of (25R)26-HC in CSF (0.14 ± 0.07 ng/mL, *P* < 0.05) compared to CP (0.10 ± 0.03 ng/mL, Fig. 2, (see also Online Resources 5 and 9). (25R)26-HC can be metabolised in the CNS to 7αH,3O-CA (Fig. 4) [29, 30]. We find that the concentration of this acid is elevated in RRMS CSF (27.59 ± 12.93 ng/mL, *P* < 0.01) compared to CP (17.40 ± 4.63 ng/mL), as is the level of its precursor 3β-hydroxycholest-5-enoic acid (3β-HCA, 1.52 ± 0.85 ng/mL cf. 0.96 ± 0.42, *P* < 0.05). The intermediary metabolite between these two acids, 3β,7α-dihydroxycholest-5-enoic acid (3β,7α-diHCA), is also elevated (3.71 ± 4.07 ng/mL cf. 2.12 ± 1.65 ng/mL) but not to significance. MS is a demyelinating disease and it likely that cholesterol is released in the CNS is metabolised through (25R)26-HC and 3β-HCA to 7αH,3O-CA, although the actual concentration of non-esterified cholesterol in CSF does not differ from controls (1.24 ± 0.33 μg/mL). 3β,7β-Dihydroxycholest-5-enoic acid (3β,7β-diHCA) which may be a metabolic product of 3β-HCA, 3β,7α-diHCA or 7-oxocholesterol (7O-C) is also elevated in CSF from RRMS patients (0.62 ± 0.35 ng/mL cf. 0.40 ± 0.19 ng/mL, *P* < 0.05).

The CSF from the ALS group shows differences in cholesterol metabolite concentrations compared to controls and also in the concentration of cholesterol itself (1.66 ± 0.36 μg/mL cf. 1.24 ± 0.33 ng/mL, *P* < 0.01). The concentration of 7α,(25R)26-dihydroxycholest-4-en-3-one (7α,(25R)26-diHCO, 0.03 ± 0.01 ng/mL, cf. 0.02 ± 0.01 ng/mL, *P* < 0.01) is increased as is its downstream metabolite 7αH,3O-CA, although not quite to significance (24.45 ± 11.16 ng/mL, cf. 17.40 ± 4.63 ng/mL, *P* = 0.07). Other neurodegenerative diseases including AD and PD are also believed to result in increased cholesterol release in brain as neurons die [28]. Björkhem and colleagues have found that patients with PD or AD can have higher CSF levels of (25R)26-HC than the controls [28, 31, 32]. We find increased concentrations of metabolites of (25R)26-HC but not of the oxysterol itself i.e. concentrations of 3β-HCA (1.57 ± 0.55 ng/mL cf. 0.96 ± 0.42 ng/mL, *P* < 0.05), 7αH,3O-CA (25.53 ± 9.52 ng/mL cf. 17.40 ± 4.63 ng/mL, *P* < 0.05) and 3β,7β-diHCA (0.64 ± 0.16 ng/mL, cf. 0.40 ± 0.19 ng/mL, *P* < 0.01) are elevated in this disease group.

Patients diagnosed with PBI show an increase in concentration of 7α,(25R)26-diHCO (0.05 ± 0.03 ng/mL cf. 0.02 ± 0.01 ng/mL, *P* < 0.05) in CSF while those diagnosed with SA/UA show increased concentrations of (25R)26-HC (0.30 ± 0.26 ng/mL cf. 0.10 ± 0.03 ng/mL, *P* < 0.05), 7α,(25R)26-dihydroxycholesterol (7α,(25R)26-diHC, 0.01 ± 0.01 ng/mL cf. 0.00 ± 0.00 ng/mL, *P* < 0.05) and 7α,(25R)26-diHCO (0.04 ± 0.02 ng/mL cf. 0.02 ± 0.01 ng/mL, *P* = 0.01).

The limit of quantification of our analytical method in CSF for most analytes is 0.01 ng/mL (10:1, signal to noise); however, for 25-HC, it is somewhat higher at 0.03 ng/mL, although detection can be made at 0.01 ng/mL. Only in the patient group of SA/UA (0.06 ± 0.04 ng/mL, cf. 0.03 ± 0.02 ng/mL, *P* < 0.05) did any of the disease states show any statistical differences in the level of 25-HC in CSF; this is also true for the 25-HC metabolite 7α,25-diHCO (0.07 ± 0.03 ng/mL cf. 0.04 ± 0.02 ng/mL, *P* < 0.05) (Fig. 3). The level of non-esterified cholesterol in control CSF is 1.24 ± 0.33 μg/mL. It is found to be elevated in the PBI group (3.44 ± 2.05 μg/mL *P* < 0.05).

## Discussion

### 25-HC Is Reduced in RRMS Plasma

In the current study, we have measured the levels of non-esterified sterols, oxysterols, 25-D_3_ and of cholestenoic and cholenoic acids in plasma and CSF of patients with MS, neurodegenerative and inflammatory CNS disease. We find that the level of 25-HC is significantly changed (*P* < 0.01) in plasma from patients with RRMS, falling from 1.54 ± 0.42 ng/mL in CP to 1.19 ± 0.26 ng/mL in RRMS plasma (Figs. 1b, e and 3). This suggests reduced transcription/translation of the gene *CH25H* in macrophages, reduced activity of the CH25H enzyme, or alternatively, enhanced clearance or metabolism of 25-HC in RRMS patients. The latter possibility is unlikely as there was no increase in concentration of downstream metabolites in plasma. 25-HC levels were also reduced, but to a lesser extent (*P* < 0.05), in plasma from patients with PBI and AD or PD. Non-esterified oxysterols are present in CSF at pg/mL levels [21], and in control subjects 25-HC is at our limit of quantification (0.03 ng/mL) and was not found to change in any of the patient groups except those with SA/UA where the level increased to 0.06 ± 0.04 ng/mL (*P* < 0.05). The plasma data for RRMS patients, indicating a reduced capacity of macrophages to synthesise 25-HC, is in-line with the recent study by Reboldi et al. which shows that in mouse macrophages 25-HC is a mediator of negative feedback towards interleukin 1 (IL-1) family cytokine production and inflammasome activity, through binding to INSIG (insulin-induced gene) and antagonising the sterol response element-binding protein-2 (SREBP-2) driven mevalonate pathway, thereby, reducing *Il1b* transcription and repressing IL-1-activating inflammasomes [33]. Thus, reduced amounts of 25-HC lead to less negative feedback towards IL-1 family cytokine production in the macrophage, with the consequent enhancement of inflammation. In keeping with these actions of 25-HC, *Ch25h* knockout mice show exacerbated experimental autoimmune encephalomyelitis (EAE), an IL-17-driven inflammatory disease model of MS, and increased susceptibility to septic shock. *Ch25h-*deficient macrophages produce more proinflammatory IL-17A^+^ T cells following lipopolysaccharide (LPS) stimulation and cytokines that cooperate with transforming growth factor-β (TGFβ) to induce T_h_17 cells include IL-1β. Notably, after LPS stimulation *Ch25h*-deficient macrophages overproduce *Il1b* [33]*.*

The mechanism of action by which reduced SREBP processing correlates with a reduction in *Il1b* transcription is not known. Reboldi et al. suggested that this may be through reducing the cellular content of sterols generated via the mevalonate pathway [33]. The results of Reboldi et al. are in contrast to those of Chalmin et al. who reported that inactivation of the *Ch25h* gene significantly attenuates EAE by limiting trafficking of pathogenic CD4+ T lymphocyte to the CNS [34]. Clearly, more work is needed to reconcile these divergent results in the literature. Although (25R)26-HC and 7αH,3O-CA were, like 25-HC, also found to be reduced (*P* < 0.05) in RRMS plasma (Fig. 1), the latter is not known to bind to INSIG, while neither sterol is formed by IFN-activated macrophages to any significant extent [8]. In contrast, 25-HC is formed by macrophages in response to IFN [7, 9, 35] and macrophages are important effector cells involved in the pathogenesis of demyelination in MS. 25-HC is also an agonist to the LXRs [36], as is (25R)26-HC but to a lesser extent and not at all in neuronal cells [21]. Besides being regulators of lipid metabolism, LXRs have also been found to modulate immune and inflammatory responses in macrophages [37]. Thus, reduced LXR activation, as a consequence of diminished 25-HC, may also explain an enhanced inflammatory response in RRMS. The involvement of LXR in the aetiology of MS is further supported by the recent report by Meffre et al. showing that LXRs are involved in the myelination and remyelination processes in oligodendrocytes [38]. In fact, LXRα or LXRβ when activated by 25-HC stimulates myelin gene expression at the promotor, mRNA and protein levels, directly implicating LXRs in the transcriptional control of myelin gene expression [38]. In addition, Wang et al. have found an LXRα mutation, in two multi-incident families presenting with severe and progressive MS disease, that disrupts LXRα heterodimerisation and transcriptional activation of target genes, further linking LXR and its agonists with MS pathogenesis [39]. In summary, a reduced capacity of macrophages to generated 25-HC may result in enhanced activity of the mevalonate pathway leading to over production of *Il1b*, and to reduced LXR activation which will both lead to enhanced inflammatory activity of macrophages recruited to the CNS in MS. It might be expected that similar to the situation in plasma, 25-HC concentrations are reduced in CSF from RRMS patients; however, 25-HC concentrations in control and RRMS patients were near the limit of quantification making statistical evaluation difficult.

### Demyelination Leads to Activation of Bile Acid Biosynthesis

In contrast to plasma, there is a significant elevation in (25R)26-HC (*P* < 0.05) and of its metabolites 3β-HCA (*P* < 0.05) and 7αH,3O-CA (*P* < 0.01) in CSF of RRMS patients (Fig. 2). These cholesterol metabolites represent early members of the “acidic pathway” of bile acid biosynthesis [40] which is known to be operative in the CNS [29, 30]. A latter member of this pathway, 7α-hydroxy-3,24-*bis*oxocholest-4-enoic acid (7αH,3,24-diO-CA), formed in the peroxisome as a thioester is also found to be elevated in RRMS CSF (Fig. 4). MS is a demyelinating disease with the consequent release of non-esterified cholesterol in the CNS. Cholesterol is metabolised in the CNS in astrocytes through cytochrome P450 (CYP) 27A1 catalysed oxidation to (25R)26-HC and subsequently to 3β-HCA and then on to 3β,7α-diHCA and 7αH,3O-CA by the consecutive action of CYP7B1 and hydroxysteroid dehydrogenase (HSD) 3B7 enzymes (Fig. 4) [41]. An increase in the availability in CNS of non-esterified cholesterol, the primary CYP27A1 substrate, is the likely explanation for the elevated activity of this pathway. Patients with CIS, which is MS resulting from a single episode of demyelination in one area of the CNS, also show an increase in the CYP27A1 product 3β-HCA (*P* < 0.05) but not its downstream metabolites. Interestingly, both 3β-HCA and 3β,7β-diHCA have been shown to be neurotoxic [21].

Patients with neurodegenerative disease including AD and PD, or ALS also show an elevation in the metabolites of the “acidic pathway” in CSF. The cholesterol metabolites 3β-HCA (*P* < 0.05) and 7αH,3O–CA (*P* < 0.01) of the “acidic pathway” are elevated in the AD and PD group, while in the ALS group 7α,(25R)26-diHCO (*P* < 0.01) is increased (Fig. 4). There are very few other studies of cholestenoic acids in CSF; however, Saeed et al. found that levels of 7αH,3O-CA were similar in AD patients and controls [42]. Brown et al. have found that CYP27A1 expression increases in oligodendrocytes in AD; this may provide an additional route to 3β-HCA from cholesterol released by dying neurons in this disease [43].

Patients with inflammatory CNS disease include those diagnosed with SA/UA and those diagnosed with PBI. The patients with SA/UA show elevated levels of 25-HC in CSF (*P* < 0.05, Fig. 3) but not in the circulation. Notably, the metabolite of 25-HC, 7α,25-diHCO is also elevated in CSF of these patients (*P* < 0.05) as are (25R)26-HC (*P* < 0.05), 7α,(25R)26-diHC (*P* = 0.05) and 7α,(25R)26-diHCO (*P* = 0.01). When plasma was analysed from these patients, none of the metabolites showed statistical differences compared to controls except 7α,(25R)26-diHCO, which unlike the situation in CSF was reduced in concentration in plasma (*P* < 0.05). Little is known of the regulation of enzymes of the acidic pathway of bile acid biosynthesis [44]. However, the drive for an increased flux through the “acidic pathway” may be a consequence of increased availability of non-esterified cholesterol. In fact, the absence of a change in non-esterified cholesterol levels in CSF in all disease groups, except those with PBI or ALS, highlights its tight regulation in CNS. The increased CSF concentrations of metabolites of the “acidic pathway” of bile acid biosynthesis indicate that this pathway represents a salvage route for removal of excess cholesterol in CNS disease which may be defective or overstretched in ALS and PBI. The explanation for the increase in 25-HC in CSF of SA/UA patients may be an up-regulation of the IFN-stimulated gene *CH25H* in macrophages of the inflamed CNS.

### Pair-Wise Correlations between Sterols

**Fig. 5:**
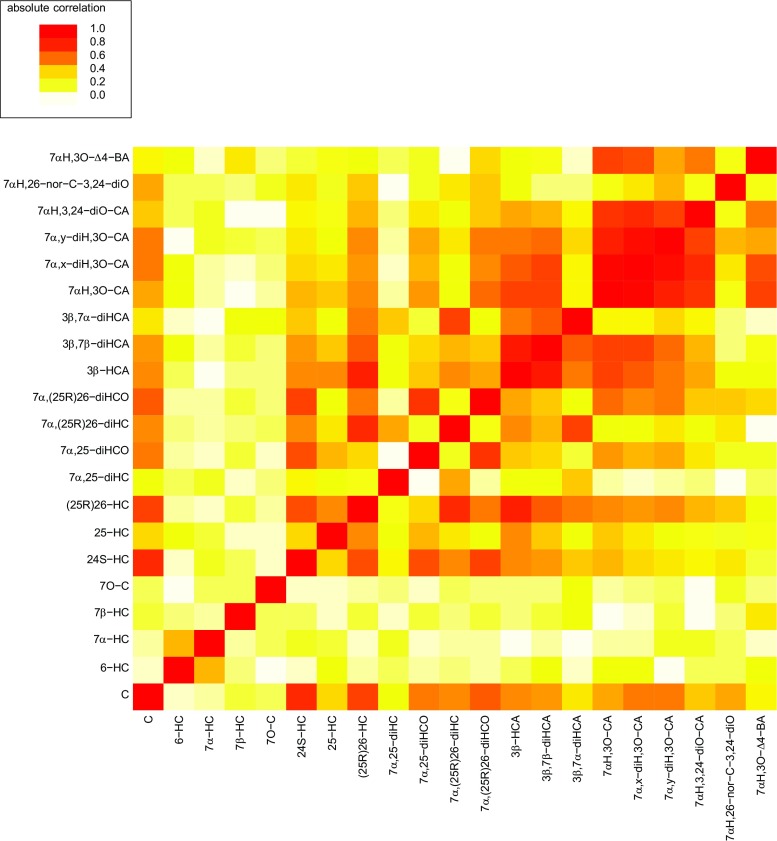
Pair-wise correlations between CSF concentration and specific analyte. The inflammatory CNS disease groups SA/UA and PBI are excluded. The Table in Online Resource 6 lists *P* values for the significance of the correlations. The *P* values that are below 0.05/((21*20)/2) = 0.000238 are highlighted in the Table; these are significant after a Bonferronni correction at 5 %. Sample numbers (*n*) are as in Fig. 1

It is of interest to study pair-wise correlations between CSF concentrations and specific sterol analytes which are arranged in metabolic order in Fig. 5. We have omitted the inflammatory CNS disease group from Fig. 5 as many correlations are driven by this group (see Online Resource 6 for *P* values for the significance of correlation (A) excluding and (B) including the inflammatory CNS disease group). As might be expected (25R)26-HC is highly correlated with 7α,(25R)26-diHC and 3β-HCA corresponding to the first metabolites of the two branches of the “acidic pathway” of bile acid biosynthesis (Fig. 4). (25R)26-HC also correlates with 7α,(25R)26-diHCO the downstream metabolite of 7α,(25R)26-diHC and with 3β,7α-diHCA and 3β,7β-diHCA, downstream metabolites of 3β-HCA, but to a lesser extent. Concentrations of (25R)26-HC also correlate with 24S-HC, presumably as both have cholesterol as their precursor. Both 24S-HC and (25R)26-HC correlate strongly with cholesterol. 25-HC also correlates with (25R)26-HC but less strongly than 24S-HC. 7α,(25R)26-diHC correlates strongly with its downstream metabolite 3β,7α-diHCA, while 7α,(25R)26-diHCO correlates most strongly with 7α,25-diHCO, both products of HSD3B7 metabolism. 3β-HCA correlates very strongly with 3β,7β-diHCA and less strongly with 7αH,3O-CA and 3β,7α-diHCA. 3β,7α-diHCA correlates most strongly with 7α,(25R)26-diHC and 3β,7β-diHCA. This data suggest that the 7α,(25R)26-diHC branch provides the primary route to 3β,7α-diHCA in CNS. 7αH,3O-CA correlates with 7αH,3,24-diO-CA and the ultimate metabolite found in CSF, 7α-hydroxy-3-oxochol-4-enoic acid (7αH,3O-Δ^4^-BA). A fascinating feature of the pair-wise correlations is the tight cluster indicated by the “red box” incorporating 7αH,3O-CA, two dihydroxy-3-oxocholest-4-enoic acid isomers (7α,x-diH,3O-CA and 7α,y-diH,3O–CA) and 7αH,3,24-diO-CA. We do not have an authentic standard for the latter compound whose identification is based on exact mass, retention time and multistage fragmentation (*MS* ^*n*^) spectrum and the location of the hydroxy groups on the two dihydroxy-3-oxocholest-4-enoic acid isomers is unclear. In both isomers, one hydroxy group is located at the 7α position while the second hydroxy group is most likely on the side chain. The pair-wise correlations between successive members of the “acidic pathway” provide strong evidence for the activity of this pathway in the CNS. The absence of correlations with 7α-hydroxycholesterol (7α-HC) confirms that the “neutral pathway” of bile acid biosynthesis is not operative in the CNS. The rate limiting enzyme of this pathway, CYP7A1, is liver specific [40]. The correlations between specific oxysterols and individual samples, including the inflammatory CNS disease group, are shown in the Fig. in Online Resource 10. As is evident from the dendrogram, the five acids 7αH,3O-Δ^4^-BA, 7αH,3,24-diO-CA; 7αH,3O-CA; 7α,x-diH,3O-CA; and 7α,y-diH,3O-CA cluster together, as do 3β-HCA, (25R)26-HC, 3β,7β-diHCA, 3β,7α-diHCA and 7α,(25R)26-diHC. The clustering patterns evident in the dendrogram in Online Resource 10 provide further evidence for an active bile acid biosynthesis pathway in CNS (Fig. 4). The dendrogram groups together 7O–C, 6-hydroxycholeserol (6-HC) and 7α-HC, these are all potential autoxidation products (see Online Resource 3).

While the metabolite correlations in CSF are dominated by the “acidic pathway” of bile acid biosynthesis, the correlations in plasma are reflective of both the “acidic pathway” and of the “neutral pathway” starting with 7α-HC followed by 7α-hydroxycholest-4-en-3-one (7α-HCO, Fig. 4) which are strongly correlated (Online Resource 11, see Online Resource 7 for *P* values for the significance of correlation). As expected from the pathway outlined in Fig. 4, 7α-HCO correlates with 7α,(25R)26-diHCO, 7αH,3OCA and 7αH,3O-Δ^4^-BA, while (25R)26-HC correlates with 3β-HCA and 7αH,3O-CA. Unsurprisingly, 3β-HCA correlates strongly with 3β,7α-diHCA, while 7αH,3O-CA correlates strongly with 7αH,3O-Δ^4^-BA. As evident from the dendrogram in the Fig. in Online Resources 12, 25-HC clusters most strongly with 24S-HC and both cluster with their precursor cholesterol, while 7α,25-diHC and 7α,(25R)26-diHC cluster together as do their HSD3B7 products 7α,25-diHCO and 7α,(25R)26-diHCO. 7α,25-diHC and 7α,(25R)26-diHC share CYP7B1 as the ultimate enzyme in their biosynthesis.

In summary, we have analysed plasma and CSF from patients with CIS and RRMS as well as from patients with inflammatory CNS disease, neurodegenerative disease and symptomatic control patients. In plasma we find that the concentration of 25-HC in RRMS patients is significantly lower than in controls. This is consistent with the recent report of Reboldi et al. that lower concentrations of 25-HC in *Ch25h-/-* mouse plasma results in reduced negative feedback by 25-HC on IL-1 family cytokine production and an exacerbated EAE, a rodent paradigm of MS [33]. In CSF, we find that the dominating cholesterol metabolites reflect the “acidic pathway” of bile acid biosynthesis and the elevated levels of these metabolites in CNS disease is likely to reflect cholesterol release as a result of demyelination or neuron death.

## Electronic Supplementary Materials

ESM 1 (DOCX 14 kb)

ESM 2 (PPTX 109 kb)

ESM 3 (DOC 109 kb)

ESM 4 (PPTX 597 kb)

ESM 5 (PPTX 550 kb)

ESM 6 (XLSX 21 kb)

ESM 7 (XLSX 23 kb)

ESM 8 (XLSX 31 kb)

ESM 9 (XLSX 32 kb)

ESM 10 (PDF 84 kb)

ESM 11 (PDF 64 kb)

ESM 12 (PDF 54 kb)

## References

[1] Beecham AH, Patsopoulos NA, Xifara DK, Davis MF, Kemppinen A, Cotsapas C, Shah TS, Spencer C, Booth D, Goris A, Oturai A, Saarela J, Fontaine B, Hemmer B, Martin C, Zipp F, D'Alfonso S, Martinelli-Boneschi F, Taylor B, Harbo HF, Kockum I, Hillert J, Olsson T, Ban M, Oksenberg JR, Hintzen R, Barcellos LF, Agliardi C, Alfredsson L, Alizadeh M, Anderson C, Andrews R, Sondergaard HB, Baker A, Band G, Baranzini SE, Barizzone N, Barrett J, Bellenguez C, Bergamaschi L, Bernardinelli L, Berthele A, Biberacher V, Binder TM, Blackburn H, Bomfim IL, Brambilla P, Broadley S, Brochet B, Brundin L, Buck D, Butzkueven H, Caillier SJ, Camu W, Carpentier W, Cavalla P, Celius EG, Coman I, Comi G, Corrado L, Cosemans L, Cournu-Rebeix I, Cree BA, Cusi D, Damotte V, Defer G, Delgado SR, Deloukas P, di Sapio A, Dilthey AT, Donnelly P, Dubois B, Duddy M, Edkins S, Elovaara I, Esposito F, Evangelou N, Fiddes B, Field J, Franke A, Freeman C, Frohlich IY, Galimberti D, Gieger C, Gourraud PA, Graetz C, Graham A, Grummel V, Guaschino C, Hadjixenofontos A, Hakonarson H, Halfpenny C, Hall G, Hall P, Hamsten A, Harley J, Harrower T, Hawkins C, Hellenthal G, Hillier C, Hobart J, Hoshi M, Hunt SE, Jagodic M, Jelcic I, Jochim A, Kendall B, Kermode A, Kilpatrick T, Koivisto K, Konidari I, Korn T, Kronsbein H, Langford C, Larsson M, Lathrop M, Lebrun-Frenay C, Lechner-Scott J, Lee MH, Leone MA, Leppa V, Liberatore G, Lie BA, Lill CM, Linden M, Link J, Luessi F, Lycke J, Macciardi F, Mannisto S, Manrique CP, Martin R, Martinelli V, Mason D, Mazibrada G, McCabe C, Mero IL, Mescheriakova J, Moutsianas L, Myhr KM, Nagels G, Nicholas R, Nilsson P, Piehl F, Pirinen M, Price SE, Quach H, Reunanen M, Robberecht W, Robertson NP, Rodegher M, Rog D, Salvetti M, Schnetz-Boutaud NC, Sellebjerg F, Selter RC, Schaefer C, Shaunak S, Shen L, Shields S, Siffrin V, Slee M, Sorensen PS, Sorosina M, Sospedra M, Spurkland A, Strange A, Sundqvist E, Thijs V, Thorpe J, Ticca A, Tienari P, van Duijn C, Visser EM, Vucic S, Westerlind H, Wiley JS, Wilkins A, Wilson JF, Winkelmann J, Zajicek J, Zindler E, Haines JL, Pericak-Vance MA, Ivinson AJ, Stewart G, Hafler D, Hauser SL, Compston A, McVean G, De Jager P, Sawcer SJ, McCauley JL (2013). Analysis of immune-related loci identifies 48 new susceptibility variants for multiple sclerosis. Nat Genet.

[2] Ramagopalan SV, Dobson R, Meier UC, Giovannoni G (2010). Multiple sclerosis: risk factors, prodromes, and potential causal pathways. Lancet Neurol.

[3] Kasper LH, Shoemaker J (2010). Multiple sclerosis immunology: the healthy immune system vs the MS immune system. Neurology.

[4] Lassmann H, Bruck W, Lucchinetti CF (2007). The immunopathology of multiple sclerosis: an overview. Brain Pathol.

[5] Friese MA, Schattling B, Fugger L (2014). Mechanisms of neurodegeneration and axonal dysfunction in multiple sclerosis. Nat Rev Neurol.

[6] Inoue M, Shinohara ML (2013). The role of interferon-beta in the treatment of multiple sclerosis and experimental autoimmune encephalomyelitis—in the perspective of inflammasomes. Immunology.

[7] Bauman DR, Bitmansour AD, McDonald JG, Thompson BM, Liang G, Russell DW (2009). 25-Hydroxycholesterol secreted by macrophages in response to toll-like receptor activation suppresses immunoglobulin A production. Proc Natl Acad Sci U S A.

[8] Blanc M, Hsieh WY, Robertson KA, Kropp KA, Forster T, Shui G, Lacaze P, Watterson S, Griffiths SJ, Spann NJ, Meljon A, Talbot S, Krishnan K, Covey DF, Wenk MR, Craigon M, Ruzsics Z, Haas J, Angulo A, Griffiths WJ, Glass CK, Wang Y, Ghazal P (2013). The transcription factor STAT-1 couples macrophage synthesis of 25-hydroxycholesterol to the interferon antiviral response. Immunity.

[9] Diczfalusy U, Olofsson KE, Carlsson AM, Gong M, Golenbock DT, Rooyackers O, Flaring U, Björkbacka H (2009). Marked upregulation of cholesterol 25-hydroxylase expression by lipopolysaccharide. J Lipid Res.

[10] Liu SY, Aliyari R, Chikere K, Li G, Marsden MD, Smith JK, Pernet O, Guo H, Nusbaum R, Zack JA, Freiberg AN, Su L, Lee B, Cheng G (2013). Interferon-inducible cholesterol-25-hydroxylase broadly inhibits viral entry by production of 25-hydroxycholesterol. Immunity.

[11] Cyster JG, Dang EV, Reboldi A, Yi T (2014). 25-Hydroxycholesterols in innate and adaptive immunity. Nat Rev Immunol.

[12] Spann NJ, Glass CK (2013). Sterols and oxysterols in immune cell function. Nat Immunol.

[13] Goodwin B, Gauthier KC, Umetani M, Watson MA, Lochansky MI, Collins JL, Leitersdorf E, Mangelsdorf DJ, Kliewer SA, Repa JJ (2003). Identification of bile acid precursors as endogenous ligands for the nuclear xenobiotic pregnane X receptor. Proc Natl Acad Sci U S A.

[14] Janowski BA, Willy PJ, Devi TR, Falck JR, Mangelsdorf DJ (1996). An oxysterol signalling pathway mediated by the nuclear receptor LXR alpha. Nature.

[15] Parks DJ, Blanchard SG, Bledsoe RK, Chandra G, Consler TG, Kliewer SA, Stimmel JB, Willson TM, Zavacki AM, Moore DD, Lehmann JM (1999). Bile acids: natural ligands for an orphan nuclear receptor. Science.

[16] Wang Y, Kumar N, Crumbley C, Griffin PR, Burris TP (2010). A second class of nuclear receptors for oxysterols: regulation of RORalpha and RORgamma activity by 24S-hydroxycholesterol (cerebrosterol). Biochim Biophys Acta.

[17] Hong C, Tontonoz P (2014). Liver X receptors in lipid metabolism: opportunities for drug discovery. Nat Rev Drug Discov.

[18] Soroosh P, Wu J, Xue X, Song J, Sutton SW, Sablad M, Yu J, Nelen MI, Liu X, Castro G, Luna R, Crawford S, Banie H, Dandridge RA, Deng X, Bittner A, Kuei C, Tootoonchi M, Rozenkrants N, Herman K, Gao J, Yang XV, Sachen K, Ngo K, Fung-Leung WP, Nguyen S, de Leon-Tabaldo A, Blevitt J, Zhang Y, Cummings MD, Rao T, Mani NS, Liu C, McKinnon M, Milla ME, Fourie AM, Sun S (2014). Oxysterols are agonist ligands of RORgammat and drive Th17 cell differentiation. Proc Natl Acad Sci U S A.

[19] Hannedouche S, Zhang J, Yi T, Shen W, Nguyen D, Pereira JP, Guerini D, Baumgarten BU, Roggo S, Wen B, Knochenmuss R, Noel S, Gessier F, Kelly LM, Vanek M, Laurent S, Preuss I, Miault C, Christen I, Karuna R, Li W, Koo DI, Suply T, Schmedt C, Peters EC, Falchetto R, Katopodis A, Spanka C, Roy MO, Detheux M, Chen YA, Schultz PG, Cho CY, Seuwen K, Cyster JG, Sailer AW (2011). Oxysterols direct immune cell migration via EBI2. Nature.

[20] Liu C, Yang XV, Wu J, Kuei C, Mani NS, Zhang L, Yu J, Sutton SW, Qin N, Banie H, Karlsson L, Sun S, Lovenberg TW (2011). Oxysterols direct B-cell migration through EBI2. Nature.

[21] Theofilopoulos S, Griffiths WJ, Crick PJ, Yang S, Meljon A, Ogundare M, Kitambi SS, Lockhart A, Tuschl K, Clayton PT, Morris AA, Martinez A, Reddy MA, Martinuzzi A, Bassi MT, Honda A, Mizuochi T, Kimura A, Nittono H, De MG, Carbone R, Criscuolo C, Yau JL, Seckl JR, Schule R, Schols L, Sailer AW, Kuhle J, Fraidakis MJ, Gustafsson JÅ, Steffensen KR, Björkhem I, Ernfors P, Sjövall J, Arenas E, Wang Y (2014). Cholestenoic acids regulate motor neuron survival via liver X receptors. J Clin Invest.

[22] Crick PJ, Bentley WT, Abdel-Khalik J, Matthews I, Clayton PT, Morris AA, Bigger BW, Zerbinati C, Tritapepe L, Iuliano L, Wang Y, Griffiths WJ (2015). Quantitative charge-tags for sterol and oxysterol analysis. Clin Chem.

[23] Park K, Scott AL (2010). Cholesterol 25-hydroxylase production by dendritic cells and macrophages is regulated by type I interferons. J Leukoc Biol.

[24] Björkhem I (2013). Five decades with oxysterols. Biochimie.

[25] Lee CY, Seet RC, Huang SH, Long LH, Halliwell B (2009). Different patterns of oxidized lipid products in plasma and urine of dengue fever, stroke, and Parkinson's disease patients: cautions in the use of biomarkers of oxidative stress. Antioxid Redox Signal.

[26] Solomon A, Leoni V, Kivipelto M, Besga A, Oksengård AR, Julin P, Svensson L, Wahlund LO, Andreasen N, Winblad B, Soininen H, Björkhem I (2009). Plasma levels of 24S-hydroxycholesterol reflect brain volumes in patients without objective cognitive impairment but not in those with Alzheimer's disease. Neurosci Lett.

[27] Björkhem I, Cedazo-Minguez A, Leoni V, Meaney S (2009). Oxysterols and neurodegenerative diseases. Mol Asp Med.

[28] Björkhem I, Lovgren-Sandblom A, Leoni V, Meaney S, Brodin L, Salveson L, Winge K, Pålhagen S, Svenningsson P (2013). Oxysterols and Parkinson's disease: evidence that levels of 24S-hydroxycholesterol in cerebrospinal fluid correlates with the duration of the disease. Neurosci Lett.

[29] Meaney S, Heverin M, Panzenboeck U, Ekström L, Axelsson M, Andersson U, Diczfalusy U, Pikuleva I, Wahren J, Sattler W, Björkhem I (2007). Novel route for elimination of brain oxysterols across the blood-brain barrier: conversion into 7alpha-hydroxy-3-oxo-4-cholestenoic acid. J Lipid Res.

[30] Ogundare M, Theofilopoulos S, Lockhart A, Hall LJ, Arenas E, Sjövall J, Brenton AG, Wang Y, Griffiths WJ (2010). Cerebrospinal fluid steroidomics: are bioactive bile acids present in brain?. J Biol Chem.

[31] Leoni V, Shafaati M, Salomon A, Kivipelto M, Björkhem I, Wahlund LO (2006). Are the CSF levels of 24S-hydroxycholesterol a sensitive biomarker for mild cognitive impairment?. Neurosci Lett.

[32] Mateos L, Ismail MA, Gil-Bea FJ, Leoni V, Winblad B, Björkhem I, Cedazo-Minguez A (2011). Upregulation of brain renin angiotensin system by 27-hydroxycholesterol in Alzheimer's disease. J Alzheimers Dis.

[33] Reboldi A, Dang EV, McDonald JG, Liang G, Russell DW, Cyster JG (2014). Inflammation. 25-Hydroxycholesterol suppresses interleukin-1-driven inflammation downstream of type I interferon. Science.

[34] Chalmin F, Rochemont V, Lippens C, Clottu A, Sailer AW, Merkler D, Hugues S, Pot C (2015). Oxysterols regulate encephalitogenic CD4(+) T cell trafficking during central nervous system autoimmunity. J Autoimmun.

[35] McDonald JG, Russell DW (2010). Editorial: 25-hydroxycholesterol: a new life in immunology. J Leukoc Biol.

[36] Lehmann JM, Kliewer SA, Moore LB, Smith-Oliver TA, Oliver BB, Su JL, Sundseth SS, Winegar DA, Blanchard DE, Spencer TA, Willson TM (1997). Activation of the nuclear receptor LXR by oxysterols defines a new hormone response pathway. J Biol Chem.

[37] Zelcer N, Tontonoz P (2006). Liver X receptors as integrators of metabolic and inflammatory signaling. J Clin Invest.

[38] Meffre D, Shackleford G, Hichor M, Gorgievski V, Tzavara ET, Trousson A, Ghoumari AM, Deboux C, Nait OB, Liere P, Schumacher M, Baulieu EE, Charbonnier F, Grenier J, Massaad C (2015). Liver X receptors alpha and beta promote myelination and remyelination in the cerebellum. Proc Natl Acad Sci U S A.

[39] Wang Z, Sadovnick AD, Traboulsee AL, Ross JP, Bernales CQ, Encarnacion M, Yee IM, de Lemos M, Greenwood T, Lee JD, Wright G, Ross CJ, Zhang S, Song W, Vilarino-Guell C (2016). Nuclear receptor NR1H3 in familial multiple sclerosis. Neuron.

[40] Russell DW (2003). The enzymes, regulation, and genetics of bile acid synthesis. Annu Rev Biochem.

[41] Zhang J, Akwa Y, el-Etr M, Baulieu EE, Sjövall J (1997). Metabolism of 27-, 25- and 24-hydroxycholesterol in rat glial cells and neurons. Biochem J.

[42] Saeed A, Floris F, Andersson U, Pikuleva I, Lövgren-Sandblom A, Bjerke M, Paucar M, Wallin A, Svenningsson P, Björkhem I (2014). 7alpha-hydroxy-3-oxo-4-cholestenoic acid in cerebrospinal fluid reflects the integrity of the blood-brain barrier. J Lipid Res.

[43] Brown J, Theisler C, Silberman S, Magnuson D, Gottardi-Littell N, Lee JM, Yager D, Crowley J, Sambamurti K, Rahman MM, Reiss AB, Eckman CB, Wolozin B (2004). Differential expression of cholesterol hydroxylases in Alzheimer's disease. J Biol Chem.

[44] Szanto A, Benko S, Szatmari I, Balint BL, Furtos I, Ruhl R, Molnar S, Csiba L, Garuti R, Calandra S, Larsson H, Diczfalusy U, Nagy L (2004). Transcriptional regulation of human CYP27 integrates retinoid, peroxisome proliferator-activated receptor, and liver X receptor signaling in macrophages. Mol Cell Biol.

