# Supplementary material for: Reduced Plasma Levels of 25-Hydroxycholesterol and Increased Cerebrospinal Fluid Levels of Bile Acid Precursors in Multiple Sclerosis Patients
Source: Mol Neurobiol. 2016 Nov 23;54(10):8009–20. doi: 10.1007/s12035-016-0281-9 (PMC5684259; doi:10.1007/s12035-016-0281-9)
Supplement: Supplementary file 1 — (DOCX 14 kb) [file 12035_2016_281_MOESM1_ESM.docx]

ESM 1. Information on patients studied.

| Code | Disease | Number of patients | Mean Age ± SD (years) | Disease status at LP | Male (M) or Female (F) | Note |
| --- | --- | --- | --- | --- | --- | --- |
| 1 | CIS | n=16 | 34.2 ± 10.1 | Relapse, n=10  Stable, n=6 | M, n=4  F, n=12 | 1 |
| 2 | RRMS | n=17 | 36.8 ± 8.8 | Relapse, n=7  Stable, n=10 | M, n=4  F, n=13 | 2 |
| 3 | CP | n=18 | 37.2 ± 13.2 | -- | M, n=6  F, n=12 | 3,4 |
| 4.1 | SA/UA | n=10 | 51.8 ± 18.3 | -- | M, n=5  F, n=5 | 5 |
| 4.2 | PBI | n=9 | 54.3 ± 19.0 | -- | M, n=4  F, n=5 | 5 |
| 5.1 | AD/PD | n=9 | 70.8 ± 11.0 | -- | M, n=7  F, n=2 | 6,7 |
| 5.2 | ALS | n=11 | 64.5 ± 10.8 | -- | M, n=7  F, n=4 | 6 |

Abbreviations. AD/PD, Alzheimer’s disease or Parkinson’s disease; ALS, amyotrophic lateral sclerosis; CIS, clinically isolated syndrome; CP, control patients; LP, lumber puncture; RRMS, relapsing remitting multiple sclerosis; SA/UA, suspected autoimmune disease or of unknown aetiology; --, not applicable.

1. No patients receiving immunomodulatory treatment.

2. One patient receiving immunomodulatory treatment at the time of lumbar puncture.

3. Patients with neurological symptoms, but no objective clinical or paraclinical findings to define a specific neurological disease at the time of sampling (CSF negative for oligoclonal bands, normal blood brain barrier function, and normal cell count).

4. Ten patients suffer from headache.

5. Inflammatory CNS disease.

6. Neurodegenerative disease.

7. Five patients with diagnosed with dementia, four patients diagnosed with PD.
