# Supplementary figures and images for: Reduced Plasma Levels of 25-Hydroxycholesterol and Increased Cerebrospinal Fluid Levels of Bile Acid Precursors in Multiple Sclerosis Patients
Source: Mol Neurobiol. 2016 Nov 23;54(10):8009–20. doi: 10.1007/s12035-016-0281-9 (PMC5684259; doi:10.1007/s12035-016-0281-9)

## Slide 1
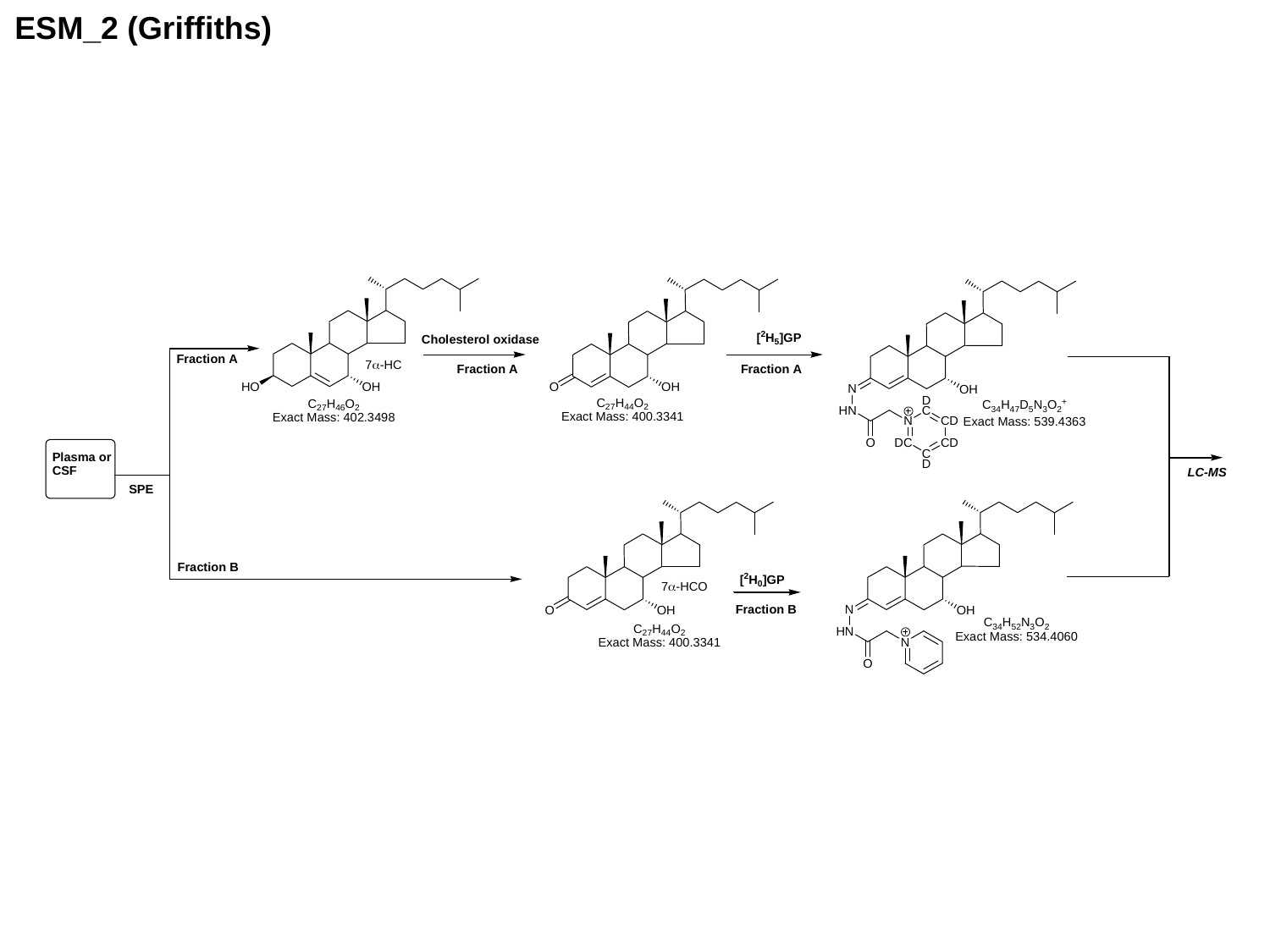

ESM_2 (Griffiths)

Supplement: Supplementary file 2 — (PPTX 109 kb) [file 12035_2016_281_MOESM2_ESM.pptx]

## Slide 1
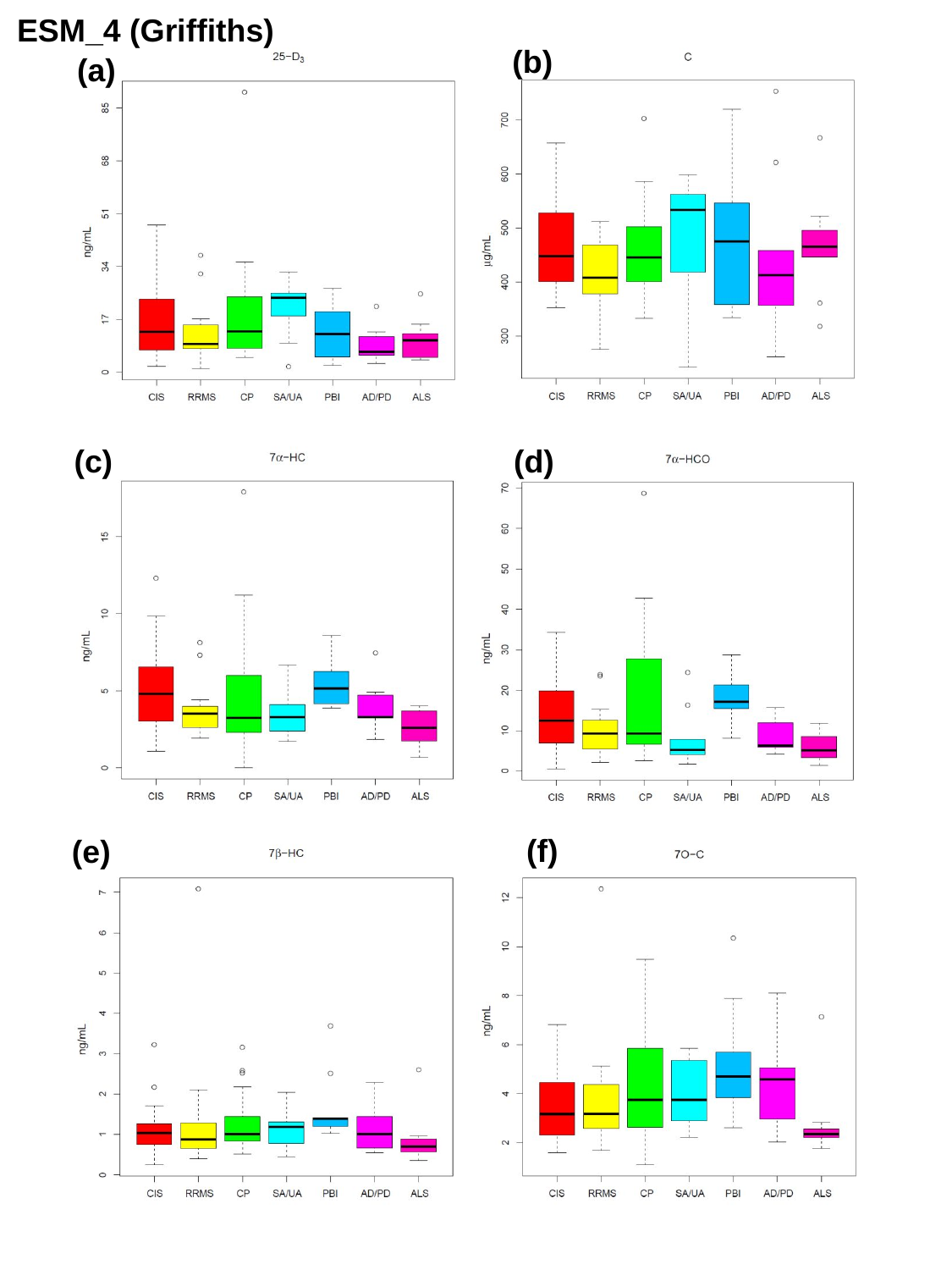

ESM_4 (Griffiths)
(b)
(a)
(c)
(d)
(f)
(e)

## Slide 2
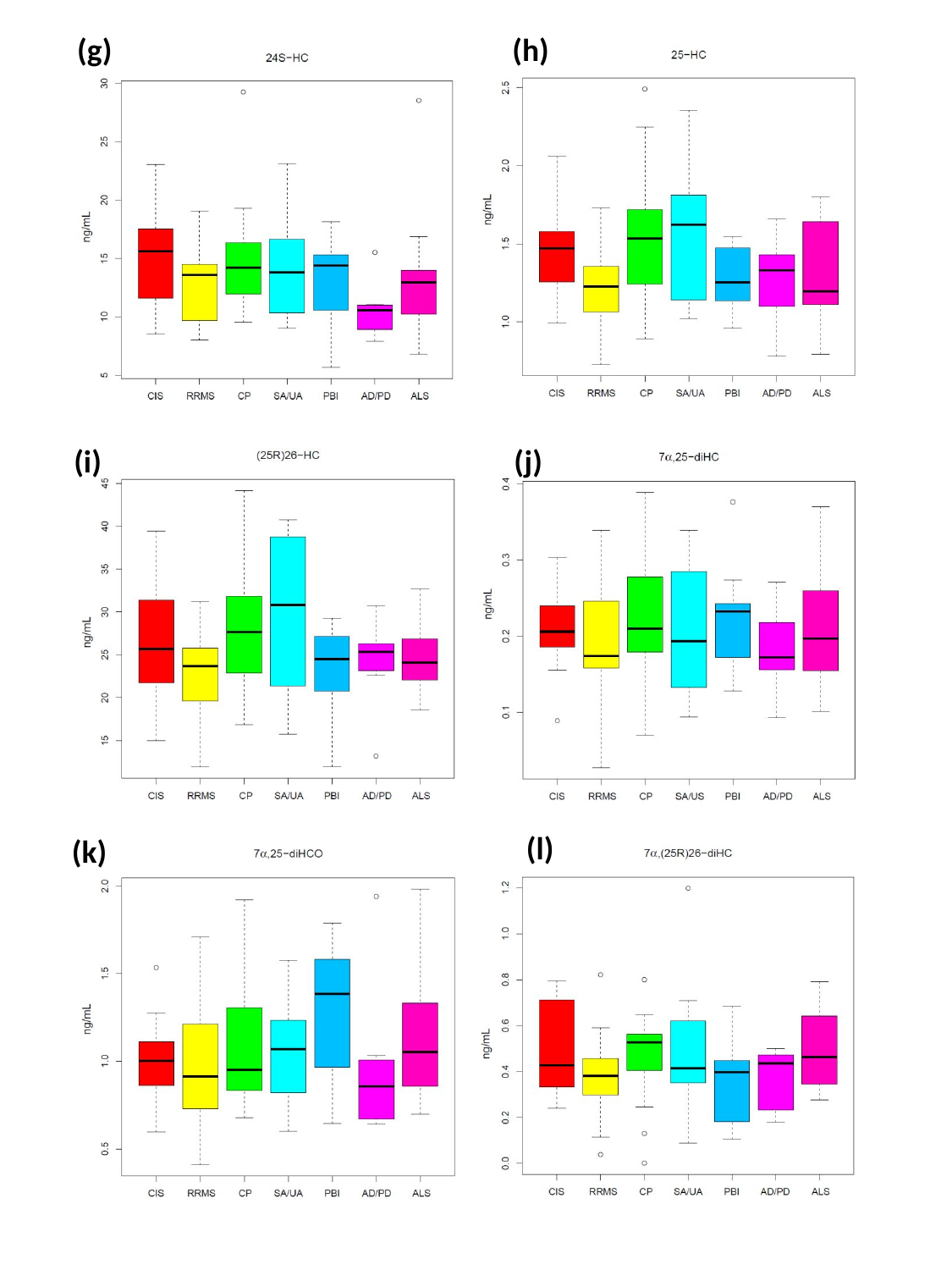

(g)
(h)
(i)
(j)
(l)
(k)

## Slide 3
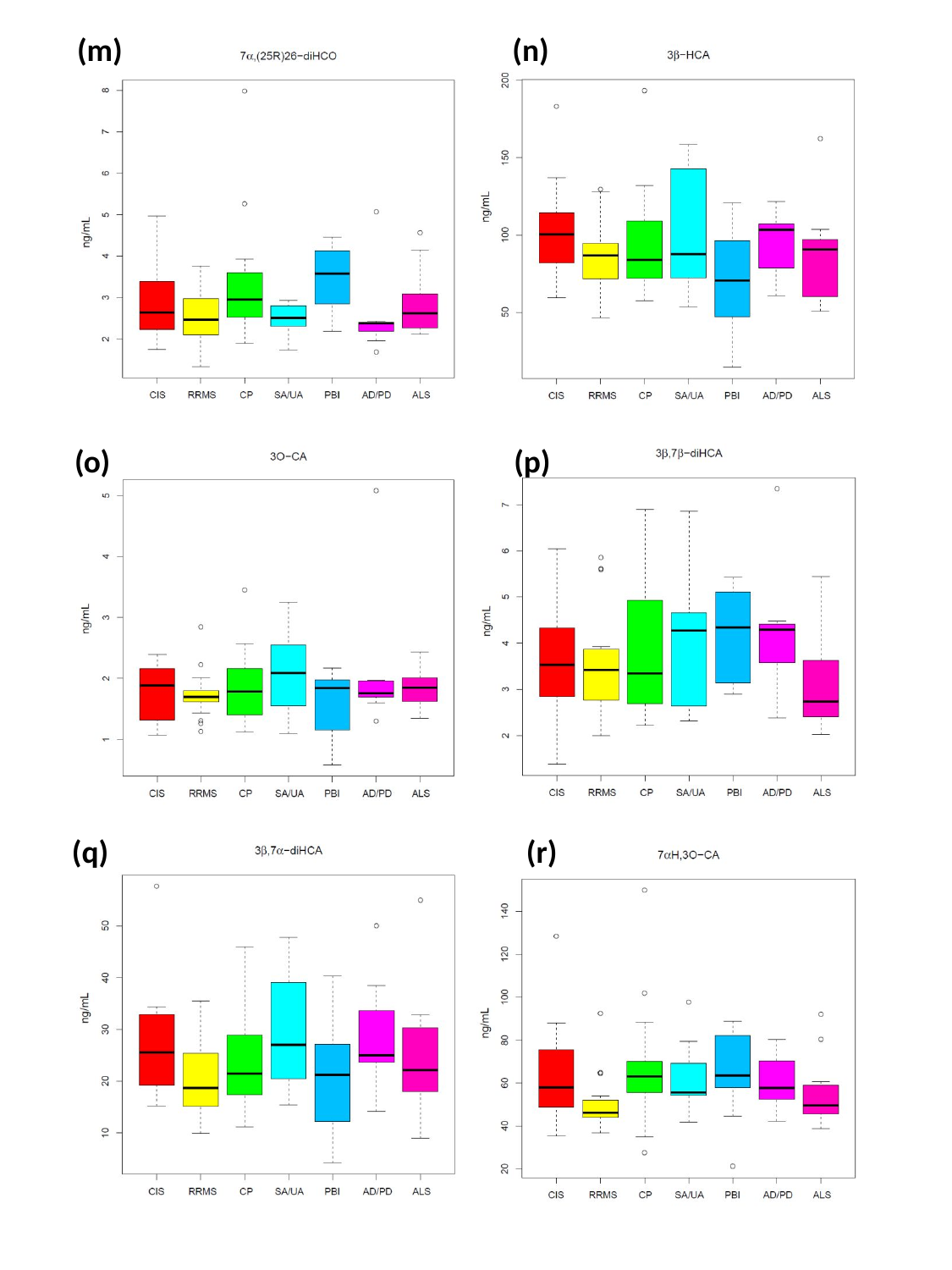

(m)
(n)
(o)
(p)
(q)
(r)

## Slide 4
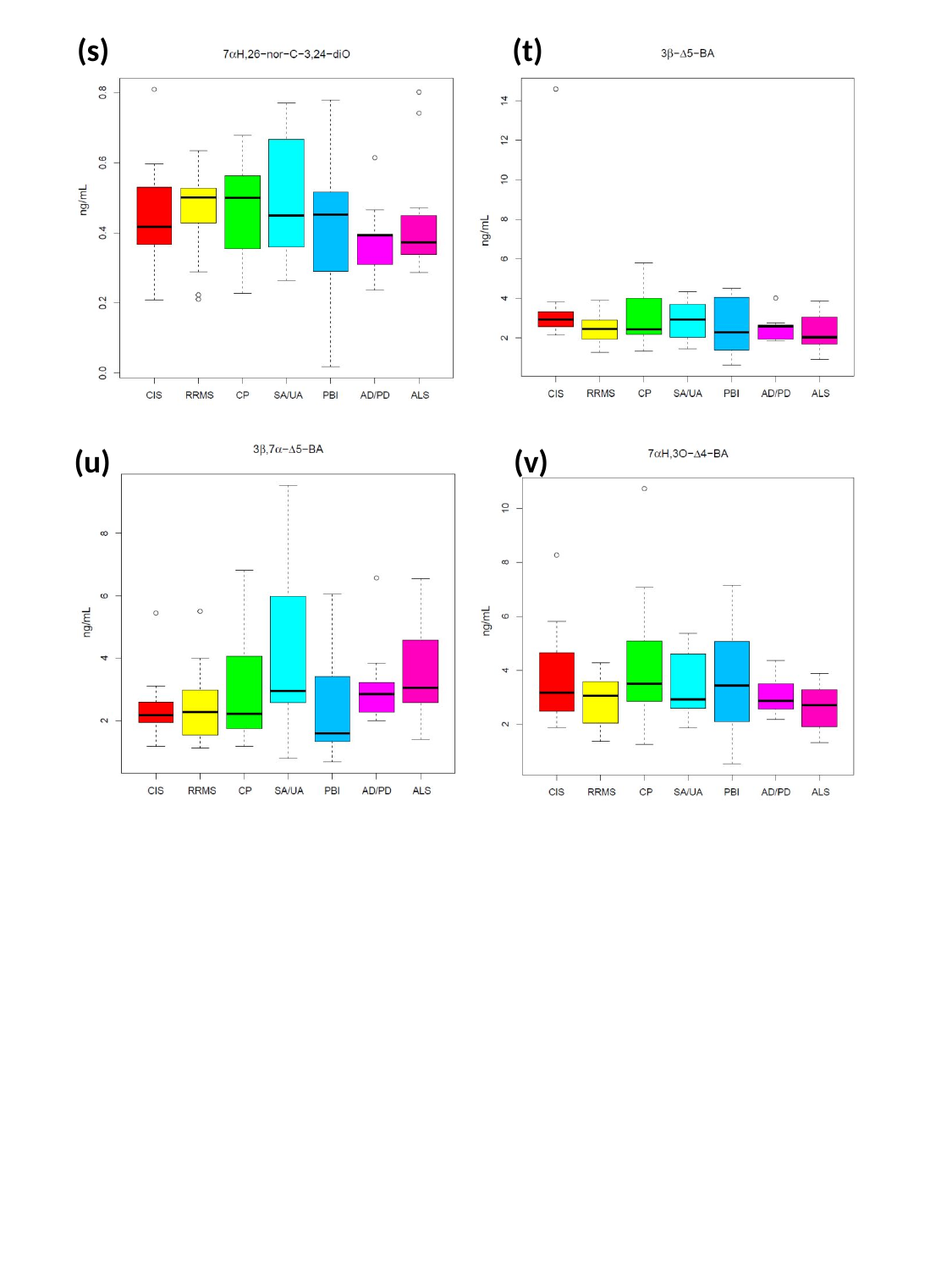

(s)
(t)
(u)
(v)

Supplement: Supplementary file 4 — (PPTX 597 kb) [file 12035_2016_281_MOESM4_ESM.pptx]

## Slide 1
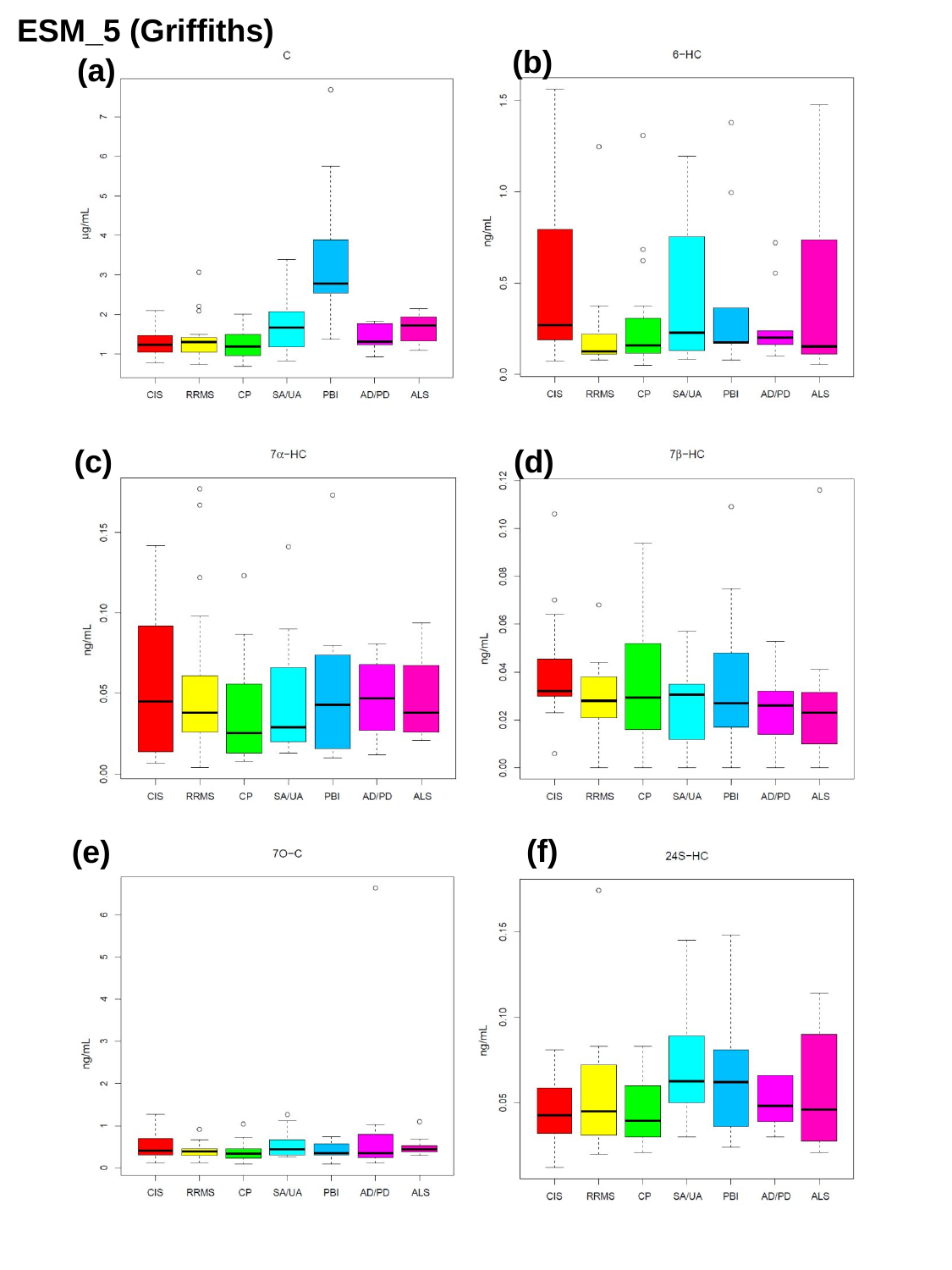

ESM_5 (Griffiths)
(b)
(a)
(c)
(d)
(f)
(e)

## Slide 2
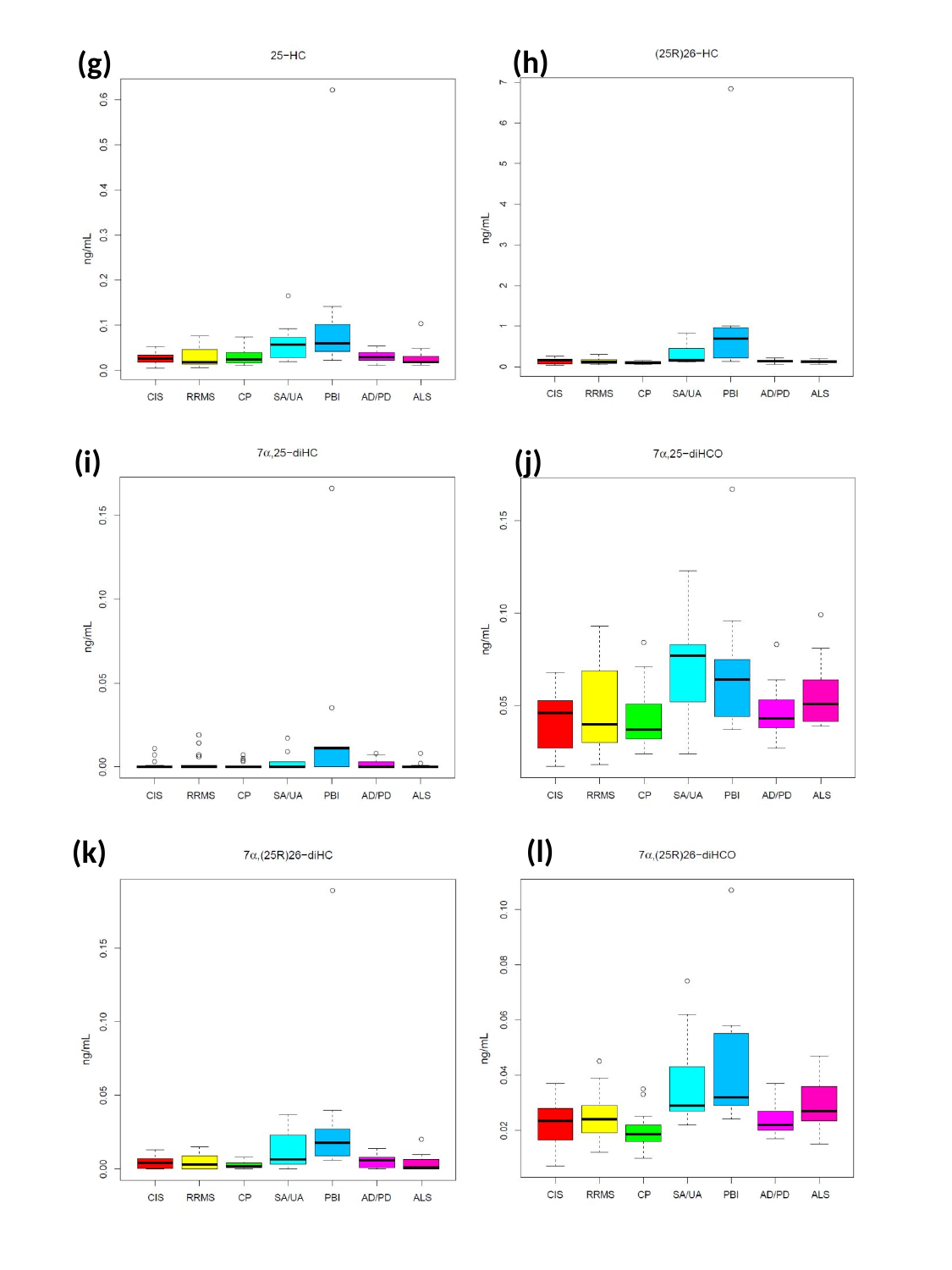

(h)
(g)
(i)
(j)
(l)
(k)

## Slide 3
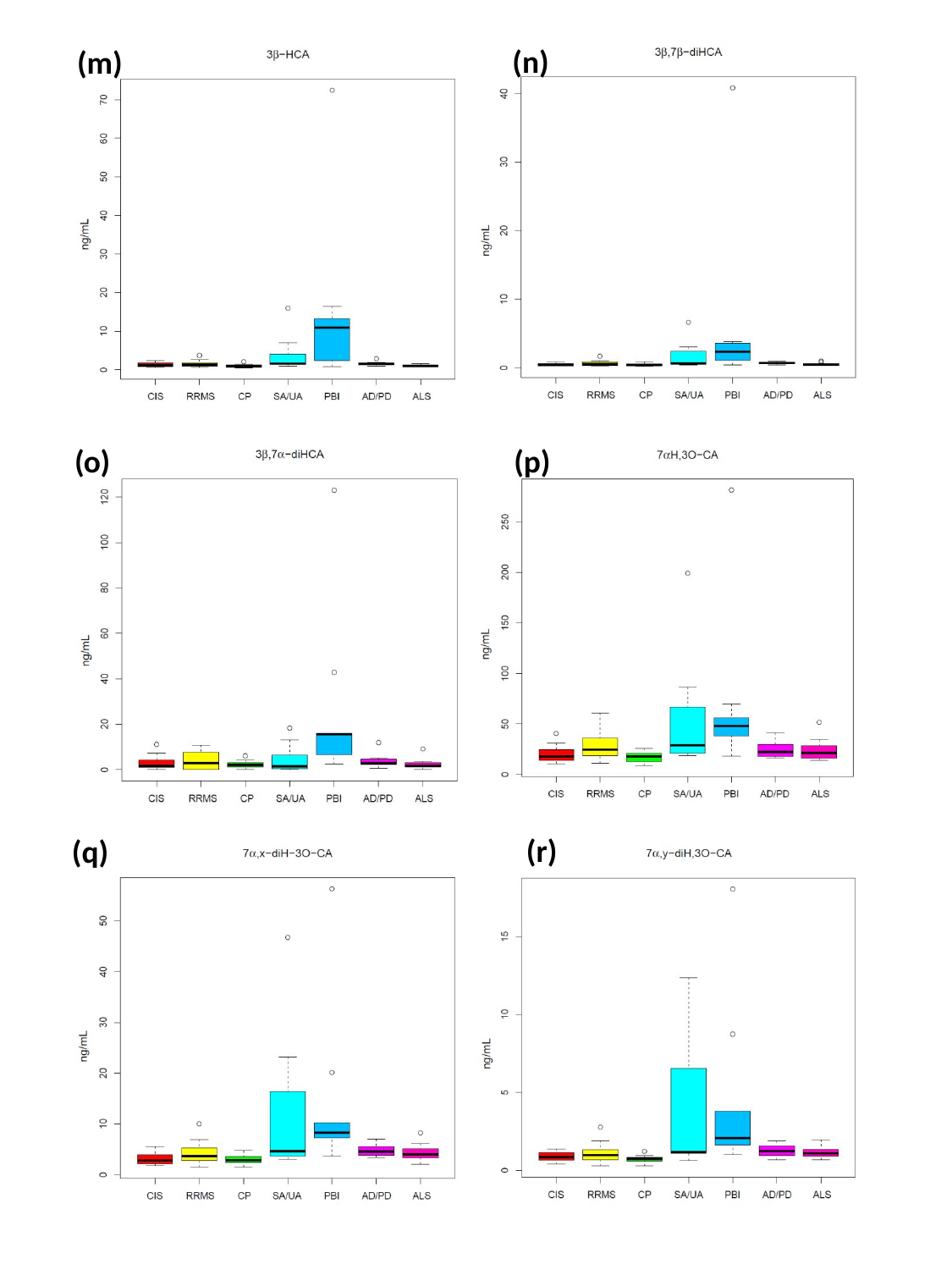

(n)
(m)
(o)
(p)
(r)
(q)

## Slide 4
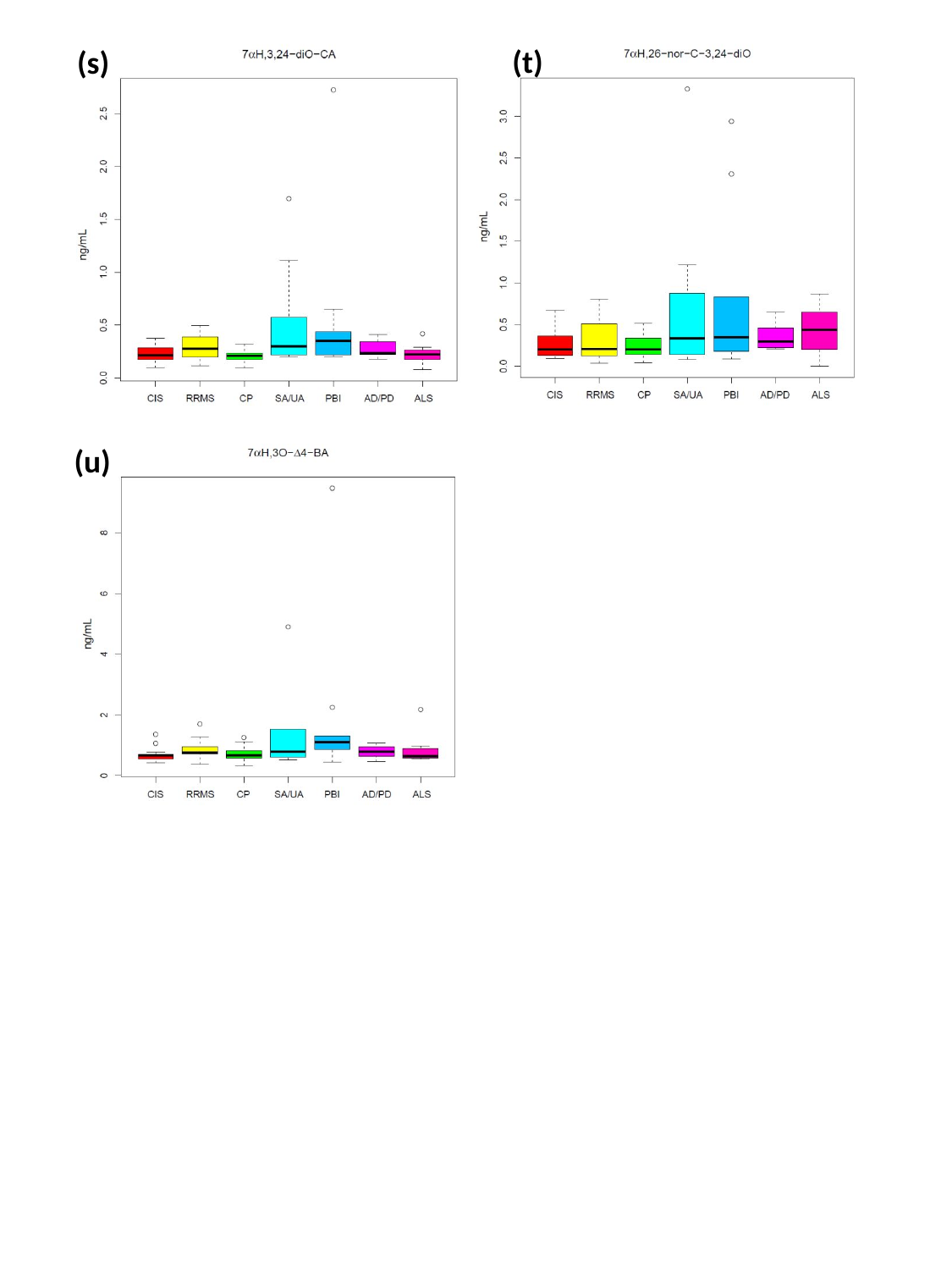

(t)
(s)
(u)

Supplement: Supplementary file 5 — (PPTX 550 kb) [file 12035_2016_281_MOESM5_ESM.pptx]

# ESM\_10 (Griffiths)

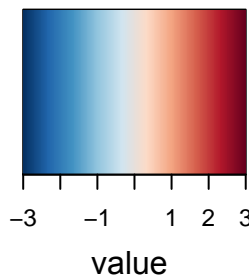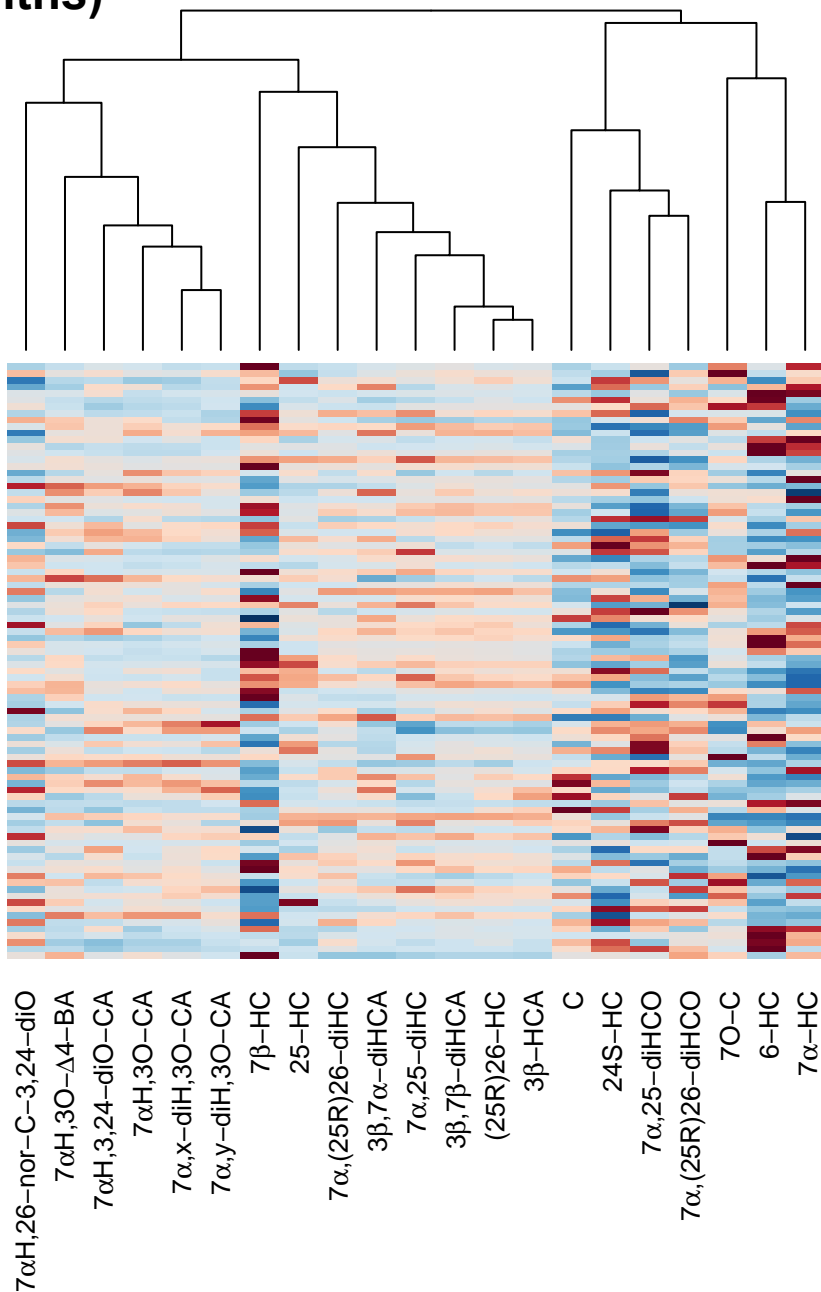

Supplement: Supplementary file 10 — (PDF 84 kb) [file 12035_2016_281_MOESM10_ESM.pdf]

## ESM\_11 (Griffiths)

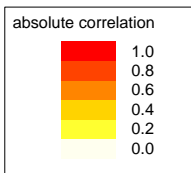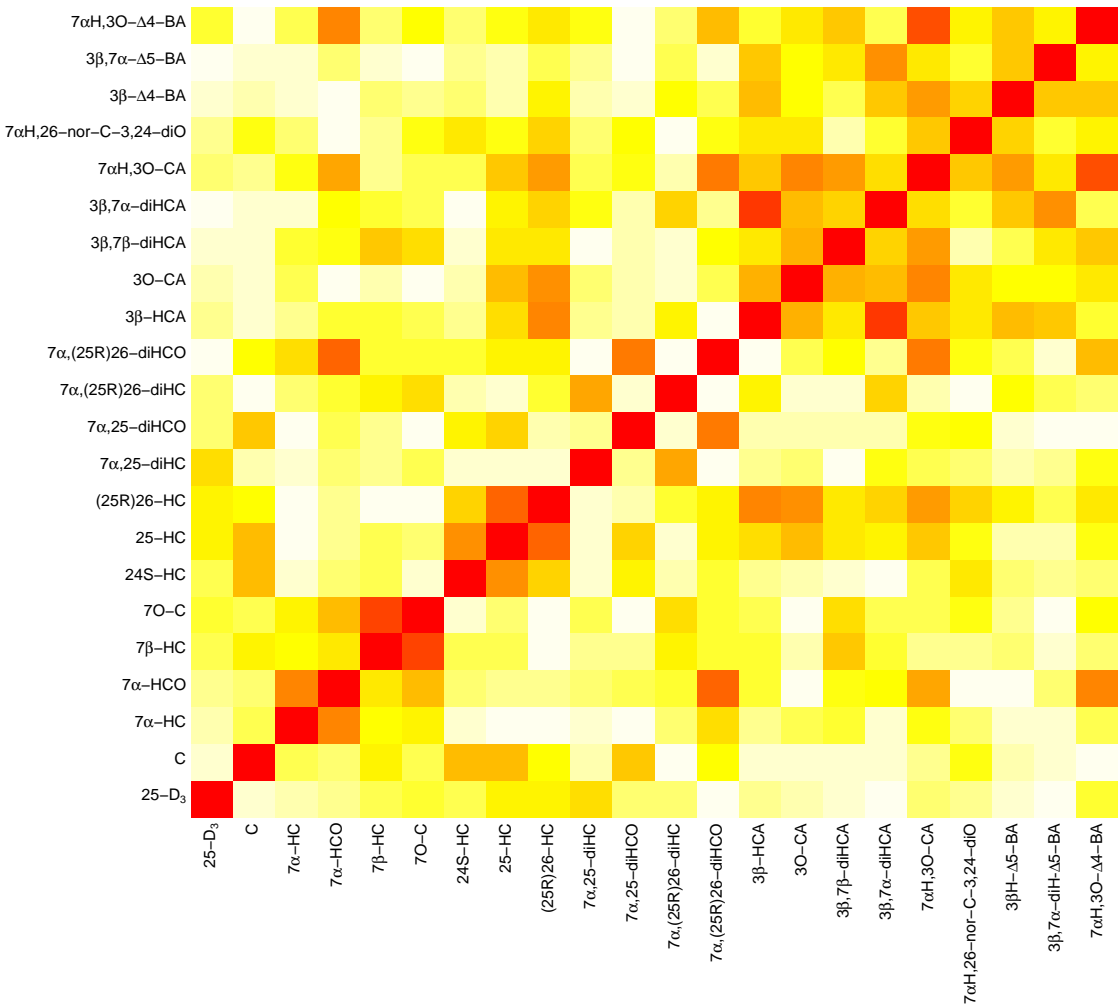

Supplement: Supplementary file 11 — (PDF 64 kb) [file 12035_2016_281_MOESM11_ESM.pdf]

ESM\_12 (Griffiths)

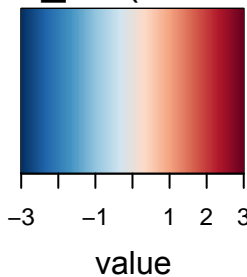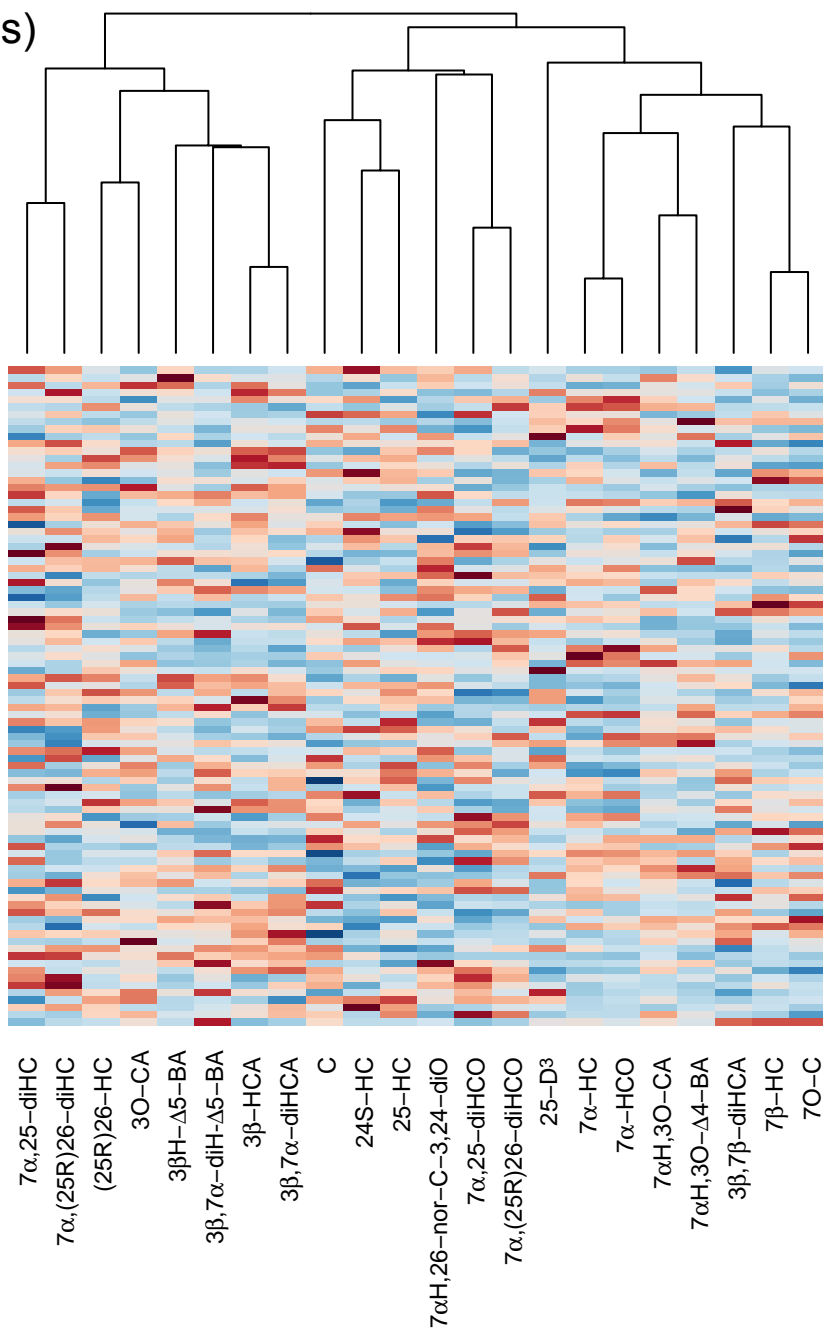

Supplement: Supplementary file 12 — (PDF 54 kb) [file 12035_2016_281_MOESM12_ESM.pdf]
