# Supplementary material for: Reduced Plasma Levels of 25-Hydroxycholesterol and Increased Cerebrospinal Fluid Levels of Bile Acid Precursors in Multiple Sclerosis Patients
Source: Mol Neurobiol. 2016 Nov 23;54(10):8009–20. doi: 10.1007/s12035-016-0281-9 (PMC5684259; doi:10.1007/s12035-016-0281-9)
Supplement: Supplementary file 3 — (DOC 109 kb) [file 12035_2016_281_MOESM3_ESM.doc]

**Reduced Plasma Levels of 25-Hydroxycholesterol and Increased Cerebrospinal Fluid Levels of Bile Acid Precursors in Multiple Sclerosis Patients**

Peter J. Crick^1^, William J. Griffiths^1^, Juan Zhang^2^, Martin Beibel^2^, Jonas Abdel-Khalik^1^, Jens Kuhle^3^, Andreas W. Sailer^4^, Yuqin Wang^1^

^1^Swansea University Medical School, Singleton Park, Swansea SA2 8PP, UK

^2^Analytical Science and Imaging, Novartis Institutes for BioMedical Research, CH-4056 Basel, Switzerland

^3^Neurology, Departments of Medicine, Biomedicine and Clinical Research, University Hospital Basel, CH-4031 Basel, Switzerland

^4^Developmental & Molecular Pathways, Novartis Institutes for BioMedical Research, CH-4056 Basel, Switzerland

**Supplementary Materials and Methods**

Lipid extraction from CSF and *LC-MS* analysis

Sterols were extracted from CSF (250 μL) into absolute ethanol (1.05 mL) containing [25,26,26,26,27,27,27-^2^H_7_]24R/S-HC (4 ng), [25,26,26,26,27,27,27-^2^H_7_]22R-hydroxycholest-4-en-3-one ([25,26,26,26,27,27,27-^2^H_7_]22R-HCO) (4 ng), [26,26,26,27,27,27-^2^H_6_]7α,25-diHC (0.4 ng) and [25,26,26,26,27,27,27-^2^H_7_]cholesterol (4 μg) (deuterated standards from Avanti Polar Lipids, Alabaster, Al, or previous studies in the laboratory) with ultrasonication. The solution was diluted with water (200 µL) to 70% ethanol, ultrasonicated for a further 5 min and centrifuged at 14,000 g at 4°C for 30 minutes. Cholesterol was removed from the supernatant by passing the 70% ethanol solution (1.5 mL) through a tC18, 200 mg Sep-Pak solid phase extraction (SPE) column (Waters, Elstree, Hertfordshire, UK) previously conditioned with absolute ethanol (4 mL) and 70% ethanol (6 mL). The column eluent combined with a column wash with 70% ethanol (5.5 mL) constitutes the oxysterol fraction (7 mL, FR1). Following a further wash with 70% ethanol (4 mL, FR2) cholesterol was eluted in absolute ethanol (2 mL, FR3). Each fraction was divided equally into A and B fractions and dried under vacuum (Online Resource 2).

Each fraction was reconstituted in propan-2-ol (50 μL). To the A fractions cholesterol oxidase (3 μL of 2 μg/μL in H_2_O, 0.044 units/μg of protein, Sigma Aldrich, Dorset, England) in 50 mM phosphate buffer pH 7 (500 μL) was added. After 1 hr at 37°C the reaction was quenched by the addition of methanol (1 mL). Fractions B were treated in an identical fashion but in the absence of cholesterol oxidase. To each fraction glacial acetic acid was added (75 μL) followed by GP reagent, [^2^H_5_]GP (85 mg, bromide salt [1]) to fractions A, [^2^H_0_]GP (75 mg, chloride salt, TCI Europe, Oxford, UK) to fractions B. After a thorough vortex the reaction was left to proceed at room temperature, overnight in the dark. Excess derivatisation reagent was then removed using a recycling procedure on a 50 mg Sep-Pak tC18 column (Waters) previously conditioned with methanol (1.5 mL), 10% methanol (1.5 mL) and 70% methanol (1.5 mL). The reaction mixture (1625 μL) was added to the column, followed by a rinse of the reaction vessel with 70% methanol (250 μL). The combined eluent was diluted with an equal volume of water and re-cycled through the column. This procedure was repeated to give an ultimate solution of 17.5% methanol which was passed through the column a final time. At this point all derivatised sterols were extracted by the column while excess reagent passed through the column. After a wash with 10% methanol (1.5 mL) derivatised sterols were eluted with methanol (0.5 mL). Each fraction was dried down and re-constituted in 60% methanol (90 μL) for *LC-MS* analysis. Fractions A and B were combined to give a final volume of 180 μL.

For oxysterol analysis 85 μL of 60% methanol solution from above, equivalent to 59 μL of CSF, was injected onto the *LC* column and MS^3^ and *MS^3^* spectra recorded. *LC* was performed on an Ultimate 3000 HPLC system (Dionex, Hemel Hempstead, Herts, UK) using a Hypersil Gold (1.9 μm, 50 x 2.1 mm) column (Thermo Fisher, Hemel Hempstead, Herts, UK). The flow rate was 200 μL/min and the eluent directed to the atmospheric pressure ionization source of an LTQ-Orbitrap Velos mass spectrometer (Thermo Fisher). The mobile phase and gradient were as described in Crick et al [1]. The LTQ-Orbitrap Velos was operated at an Orbitrap resolution of 30,000 (at *m/z* 400). A full *m/z* scan was recorded in the Orbitrap while simultaneously *MS^3^* scans were performed in the LTQ linear ion trap. The *MS^3^* transitions consisted of [M]^+^🡪[M-Py]^+^🡪. Exact *LC-MS* details can be found in Crick et al [1]. Cholesterol rich fractions were analysed in an identical fashion, however, samples were dilute by a factor of 1000 prior to injection on the *LC-MS* system.

Lipid extraction from plasma and *LC-MS* analysis

The extraction, derivatisation and analysis of sterols from plasma was essentially as described for CSF. However, the volume of plasma used was 100 μL, the amount of [^2^H_7_]24R/S-HC, [^2^H_7_]7α-HC and [^2^H_7_]22R-HCO was 20 ng, [^2^H_6_]7α,25-diHC was 2 ng and [^2^H_7_]cholesterol 20 μg, the amount of derivatisation reagent 190 mg [^2^H_5_]GP Br or 150 mg [^2^H_0_]GP Cl, and the second SPE column used for recycling was a 60 mg Oasis HLB (Waters). The procedure is described in Crick et al [1].

**Supplementary Results**

Autoxidation

7O-C is a notorious autoxidation product of cholesterol [2], although it can also be formed enzymatically from 7-dehydrocholesterol (7-DHC) [3;4]. When present at low levels, as in CSF, it is difficult to be sure that the 7O-C measured is not formed *ex vivo* via autoxidation during sample handling. We find the concentration of 7O-C in CSF to be about 0.5 ng/mL which is similar to the value reported earlier by Leoni et al [5]. Leoni et al found that 7O-C was slightly (1.2 ng/mL cf. 0.9 ng/mL) but significantly (P = 0.002) greater in MS patients than controls [5]. This data contrasts with that of Diestel et al who reported values of 7.4 μg/mL for MS patients and 500 ng/mL for other neurological disorders [6]. It is likely that there was a problem of *ex vivo* autoxidation in the study of Diestel et al [6], as such high levels of 7O-C have not been corroborated by other studies [7]. Autoxidation of cholesterol must be considered in all studies of oxysterols, as cholesterol is often 1000 times more abundant than most endogenous oxysterols [8]. Cholesterol autoxidation is the likely reason for the miss-identification of 15-oxygenated oxysterols at apparent high concentrations in serum of MS patients [9]. Farez et al reported 15-hydroxycholesterol (15-HC) concentrations in serum from MS patients in the progressive phase to be in excess of 1 μg/mL and in the RRMS phase and in controls to be about 500 ng/mL [9]. Björkhem et al could not confirm this data failing to detect 15-HC (< 2 ng/mL) in plasma of MS patients or controls [10]. The findings of Björkhem et al were further confirmed by other groups including our own [11]. In Björkhem et al’s study of oxysterols in plasma from MS patients and controls, 25-HC was found to be lower in plasma from RRMS patients than controls but not significantly [10]. An important difference between Björkhem et al’s study and that reported here is that Björkhem et al measured total oxysterol levels while we measure the levels of the unesterified molecules [10].

**Online Resource**

**ESM_1** Information on patients studied.

**ESM_2** Derivatisation of sterols, oxysterols, cholestenoic and cholenoic acids for *LC-MS* analysis. The method is exemplified for 7α-HC and 7α-HCO.

**ESM_3** Supplementary Materials and Methods and Supplementary Results.

**ESM_4** Effect of CNS disease on sterol concentrations in plasma. Box and whiskers plots showing the concentrations of cholesterol (µg/mL), oxysterols, cholestenoic acids, cholenoic acids and 25-D_3_ (all ng/mL) in plasma from CIS (n = 16), RRMS (n = 17), CP (n = 18), SA/UA (n = 10), PBI (n = 9), AD/PD (n = 9) and ALS (n = 11) patients. Online Resource 8 gives the exact concentrations for each group.

**ESM_5** Effect of CNS disease on sterol concentrations in CSF. Box and whiskers plots showing the concentrations of cholesterol (µg/mL), oxysterols, cholestenoic and cholenoic acids (all ng/mL) in CSF from CIS (n = 16), RRMS (n = 17), CP (n = 18), SA/UA (n = 10), PBI (n = 9), AD/PD (n = 9) and ALS (n = 11) patients. Online Resource 9 gives the exact concentrations for each group.

**ESM_6** P values for the significance of the pair-wise correlations between CSF concentration and specific analyte. The P values that are below 0.05/((21*20)/2) = 0.000238 are highlighted, these are significant after a Bonferronni correction at 5%.

**ESM_7** P values for the significance of pair-wise correlations between plasma concentration and analyte. The P values that are below 0.05/((22*21)/2) = 0.000216 are highlighted, these are significant after a Bonferronni correction at 5%.

**ESM_8** Concentrations of cholesterol, oxysterols, cholestenoic acids, cholenoic acids and 25-D_3_ in plasma.

**ESM_9** Concentrations of cholesterol, oxysterols, cholestenoic acids, cholenoic acids in CSF.

**ESM_10** Correlation between analyte and single CSF samples. Data for all patient groups is included. Each row corresponds to a single sample.

**ESM_11** Pair-wise correlations between plasma concentration and specific analyte including all sample groups. Online Resource 7 lists P values for the significance of the correlations. The P values that are below 0.05/((22*21)/2) = 0.000216 are highlighted in Online Resource 7, these are significant after a Bonferonni correction at 5%.

**ESM_12** Correlation between analyte and single plasma samples. Data for all patient groups is included. Each row corresponds to a single sample.

Reference List

1. Crick PJ, William BT, Abdel-Khalik J, Matthews I, Clayton PT, Morris AA, Bigger BW, Zerbinati C, Tritapepe L, Iuliano L, Wang Y, Griffiths WJ (2015) Quantitative charge-tags for sterol and oxysterol analysis. Clin Chem 61: 400-411

2. Schroepfer GJ, Jr. (2000) Oxysterols: modulators of cholesterol metabolism and other processes. Physiol Rev 80: 361-554

3. Björkhem I, Diczfalusy U, Lövgren-Sandblom A, Starck L, Jonsson M, Tallman K, Schirmer H, Ousager LB, Crick PJ, Wang Y, Griffiths WJ, Guengerich FP (2014) On the formation of 7-ketocholesterol from 7-dehydrocholesterol in patients with CTX and SLO. J Lipid Res 55: 1165-1172

4. Shinkyo R, Xu L, Tallman KA, Cheng Q, Porter NA, Guengerich FP (2011) Conversion of 7-dehydrocholesterol to 7-ketocholesterol is catalyzed by human cytochrome P450 7A1 and occurs by direct oxidation without an epoxide intermediate. J Biol Chem 286: 33021-33028

5. Leoni V, Lutjohann D, Masterman T (2005) Levels of 7-oxocholesterol in cerebrospinal fluid are more than one thousand times lower than reported in multiple sclerosis. J Lipid Res 46: 191-195

6. Diestel A, Aktas O, Hackel D, Hake I, Meier S, Raine CS, Nitsch R, Zipp F, Ullrich O (2003) Activation of microglial poly(ADP-ribose)-polymerase-1 by cholesterol breakdown products during neuroinflammation: a link between demyelination and neuronal damage. J Exp Med 198: 1729-1740

7. Leoni V, Caccia C (2013) Potential diagnostic applications of side chain oxysterols analysis in plasma and cerebrospinal fluid. Biochem Pharmacol 86: 26-36

8. Björkhem I (2013) Five decades with oxysterols. Biochimie 95: 448-454

9. Farez MF, Quintana FJ, Gandhi R, Izquierdo G, Lucas M, Weiner HL (2009) Toll-like receptor 2 and poly(ADP-ribose) polymerase 1 promote central nervous system neuroinflammation in progressive EAE. Nat Immunol 10: 958-964

10. Björkhem I, Lovgren-Sandblom A, Piehl F, Khademi M, Pettersson H, Leoni V, Olsson T, Diczfalusy U (2011) High levels of 15-oxygenated steroids in circulation of patients with multiple sclerosis: fact or fiction? J Lipid Res 52: 170-174

11. Björkhem I, Diczfalusy U, Olsson T, Russell DW, McDonald JG, Wang Y, Griffiths WJ (2011) Detecting oxysterols in the human circulation. Nat Immunol 12: 577-578
